# Dietary index for gut microbiota and risk of gastrointestinal cancer: a prospective gene-diet study

**DOI:** 10.1186/s12937-025-01151-3

**Published:** 2025-05-17

**Authors:** Dong-Run Li, Bang-Quan Liu, Ming-Hui Li, Ying Qin, Jia-Cheng Liu, Wen-Rui Zheng, Ting-Ting Gong, Shan-Yan Gao, Qi-Jun Wu

**Affiliations:** 1 Department of Clinical Epidemiology, Shengjing Hospital of China Medical University, Shenyang, China; 2 Key Laboratory of Precision Medical Research on Major Chronic Disease, Shengjing Hospital of China Medical University, Shenyang, China; 3 Department of Epidemiology, School of Public Health, China Medical University, Shenyang, China; 4 Department of Obstetrics and Gynecology, Shengjing Hospital of China Medical University, Shenyang, China; 5 NHC Key Laboratory of Advanced Reproductive Medicine and Fertility (China Medical University), National Health Commission, Shenyang, China; 6 Clinical Trials and Translation Center, Shengjing Hospital of China Medical University, Shenyang, China

**Keywords:** Dietary index for gut microbiota, Genetic risk, Gastrointestinal cancer

## Abstract

**Background:**

The dietary index for gut microbiota (DI-GM) is a newly proposed index that evaluates dietary intake patterns associated with gut microbial health. Limited studies have examined whether DI-GM influences gastrointestinal (GI) cancer risk. We aimed to investigate the association between DI-GM and GI cancer risk and evaluate its combined effect with genetic risk.

**Methods:**

We included 178,148 UK Biobank participants who completed at least one 24-hour dietary recall. DI-GM was constructed from 13 dietary components known to influence gut microbial health and was divided into three groups. The GI cancer polygenic risk score was calculated from 205 significant single-nucleotide polymorphisms related to esophageal cancer (EC), gastric cancer (GC), and colorectal cancer (CRC). Cox proportional hazards models with hazard ratios (HRs) and 95% confidence intervals (CIs) were used to estimate the associations between DI-GM, genetic risk, and GI cancer.

**Results:**

During a median follow-up of 13.47 years, 2,682 participants developed GI cancer. In fully adjusted models, higher DI-GM was associated with a lower GI cancer risk (HR for GI cancer: 0.83; 95% CI: 0.75–0.92; HR for EC: 0.62, 95% CI: 0.45–0.86; HR for GC: 0.99, 95% CI: 0.71–1.39; HR for CRC: 0.84, 95% CI: 0.75–0.95), compared with participants in the lowest DI-GM category. In joint analysis, individuals with higher DI-GM and lower genetic risk had lower GI cancer risk, with HRs (95% CI) of 0.28 (0.21, 0.36), 0.50 (0.42, 0.58) for low and intermediate genetic risk, respectively, compared with those with low DI-GM and high genetic risk. And a significant interaction between DI-GM and genetic risk was observed.

**Conclusion:**

Higher DI-GM was associated with a lower risk of GI cancer including EC and CRC. These findings highlight the importance of considering a gut microbiota-friendly diet and genetic risk in GI cancer prevention.

**Supplementary Information:**

The online version contains supplementary material available at 10.1186/s12937-025-01151-3.

## Introduction

Gastrointestinal (GI) cancer, including colorectal cancer (CRC), gastric cancer (GC), and esophageal cancer (EC), is among the most frequently diagnosed malignancies worldwide, with high incidence and mortality rates [1]. According to the Global Burden of Disease Study, GI cancers account for 26% of the global cancer incidence and 35% of all cancer-related deaths [2]. Established risk factors include age, sex, genetic predisposition, smoking, alcohol consumption, and unhealthy dietary habits [3].

In recent years, the gut microbiota has received growing attention in cancer research. It is critical in immune regulation, nutrient metabolism and inflammatory control. Emerging evidence also connects it to a range of chronic diseases, such as obesity, diabetes, cardiovascular disease, neurological disorders, and cancer [4, 5]. In particular, an imbalanced gut microbiota has been associated with tumor initiation, progression and prognosis in GI cancers [6–8]. Diet is a major determinant of the gut microbiota composition, and it is estimated that 21.5% of GI cancer cases worldwide can be attributed to suboptimal dietary practices [9]. Numerous studies have shown that certain dietary patterns (e.g., Healthy Eating diet, Mediterranean diet, Western diet, and high-fat diet) can alter the diversity and functionality of gut microbiota, thereby influencing GI cancer risk [10–12]. The dietary index for gut microbiota (DI-GM), developed by Kase et al., is a novel integrative index that captures dietary patterns conducive to a healthy gut microbiota. It has been validated by markers of gut microbiota health [13]. The development of DI-GM provides a new quantitative approach to investigate the relationships between diet, gut microbiota, and diseases. However, its role in GI cancer remains relatively underexplored.

Gene-lifestyle interaction theory suggests that the impact of modifiable lifestyle factors on disease risk may differ depending on an individual’s genetic predisposition [14]. Advances in genome-wide association studies have identified genetic variants associated with GI cancer [15–17], laying a foundation for cancer polygenic risk scores (CPRS) that reflect the cumulative influence of multiple risk alleles [18, 19]. Previous studies have shown that dietary patterns and genetic risk can jointly affect both the occurrence and progression of GI cancers [20, 21].

In this study, based on the UK Biobank database, we aimed to investigate the associations of DI-GM with GI cancer risk and further assess the joint effects of DI-GM and genetic risk on the risk of GI cancer.

## Method

### Study design and population

Data were derived from the UK Biobank, a large-scale cohort study that recruited over 500,000 adults from the United Kingdom during 2006–2010 [22]. We included 201,131 participants who completed 24-hour food recall questionnaires. We excluded participants who had been diagnosed with cancer at baseline (*n* = 17,502), had implausible total energy intake (< 800 or > 5000 kcal/day for males and < 500 or > 4000 kcal/day for females; *n* = 1,795) [23], withdrew from the study (*n* = 65), or had incomplete genetic data for constructing CPRS at baseline (*n* = 3,621). The final analytic sample comprised 178,148 participants (Supplementary Fig. 1). This study was conducted under UK Biobank application number 211,772.

### Definition of dietary index for gut microbiota

The UK Biobank employed a validated online tool for 24-hour dietary assessments (Oxford WebQ) to collect comprehensive data on the quantity and types of food consumed [24]. It has been confirmed for accuracy through comparison with an interviewer-administered 24-hour dietary recall and biomarkers [25, 26]. To estimate nutrient intake, the Oxford WebQ automatically computes total nutrient consumption by multiplying the number of portions consumed by each food’s predetermined portion size and its corresponding nutrient composition. The nutrient composition data used for these calculations were derived from the UK Nutrient Databank food composition Table (24). Initial dietary assessment took place at the assessment centers between April 2009 and September 2010, followed by four additional online questionnaires after recruitment ended. If participants completed dietary assessments multiple times, we used the means of all available measurements to calculate dietary intake.

Based on the scoring criteria of the article by Kase et al. [13], 14 food items or nutrients were identified as components of DI-GM, including avocado, broccoli, chickpeas, coffee, cranberries, fermented dairy, fiber, green tea, soybean, and whole grains as beneficial components, while high-fat diet (≥ 40% energy from fat), red meat, processed meat, and refined grains were considered adverse components. For beneficial to gut microbiota items, a score of 1 was assigned when consumption ≥ sex-specific median, otherwise 0 score; for unfavorable to gut microbiota items, a score of 0 was assigned when consumption ≥ sex specific median, otherwise 1 score. Scores were summed to obtain the overall DI-GM, ranging from 0 to 13 (chickpeas unavailable due to the UK Biobank not recording the consumption). The higher the index, the greater adherence to the beneficial gut microbiota diet. The details of scoring criteria, coding information and portion size for each component used in this study can be seen in Supplementary Tables 1–3.

### Cancer polygenic risk score for GI cancer

The genetic data from the UK Biobank have undergone extensive quality control and imputation as described previously [27]. Briefly, genotyping was performed using the UK BiLEVE array and the UK Biobank Axiom array, followed by QC measures including variant and sample-level filtering, population structure adjustment, and imputation using the Haplotype Reference Consortium panel. To evaluate the genetic predisposition of EC, GC, and CRC, we constructed a PRS based on 205 single-nucleotide polymorphisms (SNPs) that reached the genome-wide significance threshold (*P* < 5 × 10^− 8^) in populations of European ancestry [28–30]. Detailed information on the SNPs is provided in Supplementary Table 4. The calculation of PRS was performed by using the PRSice-2 algorithm with the following formula:

$$\:PR{S_i} = \sum\limits_{k = 1}^k {{\beta _k}SN{P_{i,k}}} $$

Where β_k_ value is the summary statistic for the effective allele and SNP_i, k_ is the number of the effective allele observed.

Then the CPRS was built to serve as an indicator of genetic risk for overall GI cancer, following this process:

$$\:CPR{S_i} = \sum\limits_{k = 1}^k {{h_k}PR{S_{i,k}}} $$

The cancer polygenic risk score for the i^th^ individual is denoted as PRS_i, k_, where h_k_ represents the age-standardized incidence of cancer type k in the UK population.

Based on their CPRS, participants were categorized into three genetic risk groups: low risk (lowest quintile), moderate risk (quintiles 2 to 4), and high risk (highest quintile). The details of construction of CPRS for GI cancer are provided in Supplementary Method.

### Outcome assessment

The primary outcome of interest was incident GI cancer, identified from the UK Biobank-linked national cancer registries as a first-ever diagnosis coded according to the International Classification of Diseases, 10th Revision, as esophagus (C15), stomach (C16), colorectum (C18-20). Follow-up commenced at the date of baseline assessment and ended at the earliest occurrence of any of the following events: the first recorded GI cancer diagnosis, death from any cause, or the end of follow-up on May 31, 2022. Detailed codes are provided in Supplementary Table 3.

### Covariates

Covariates were obtained from baseline data, and potential confounders were selected based on prior literature and biologically plausible associations [20, 21]. The following covariates were included: age at recruitment (continuous), sex, annual household income (categorized as <£31,000, ≥£31,000, or unknown/missing), education level (high qualifications, middle qualifications, no qualifications, or unknown/missing), and the Townsend deprivation index (continuous), a widely used measure of socioeconomic deprivation derived from participants’ residential postcodes, with lower values indicating higher socioeconomic status. Anthropometric measures, including height and weight, were collected by trained staff at baseline, and body mass index (BMI) was calculated as weight (kg)/ [height (m)] ² (continuous). Family history of cancer (yes, no, or unknown) was self-reported at baseline. Lifestyle and behavioral factors included total energy intake (continuous), smoking status (never, previous, current, or unknown/missing), alcohol consumption (never, previous, current, or unknown/missing), and physical activity (high, moderate, low). Missing data for continuous covariates were imputed using mean values, while missing categorical covariates were assigned to an unknown/missing indicator category. Detailed coding information is shown in Supplementary Table 3.

### Statistical analysis

Based on our population characteristics and previous references [31], DI-GM were classified into three groups: low (0–4), moderate [5, 6], and high (≥ 7). Baseline characteristics were described as mean with standard deviation (SD) or median with interquartile range (IQR) for continuous variables, and as counts with percentages for categorical variables, stratified by DI-GM groups. To assess differences, the Student’s t-test or Kruskal-Wallis test was applied for continuous variables, and chi-square tests for categorical variables.

The proportional hazards assumption was tested using the Schoenfeld residual test, with no violations observed (*P* > 0.05). Multivariable Cox proportional hazards regression models were conducted to assess associations between DI-GM, CPRS, and the risks of GI cancer, with results expressed as hazard ratios (HRs) and 95% confidence intervals (CIs). The models were adjusted for age, sex, BMI, total energy intake, income, education level, the Townsend deprivation index, smoking status, alcohol consumption, physical activity, and family history of cancer. The DI-GM and CPRS were analyzed by both categorical variable (low group as reference) and continuous variable (per 1-SD). Additionally, we examined the potential nonlinear relation between DI-GM, CPRS and GI cancer risk with restricted cubic splines, and the model was conducted with 4 knots at the 5th, 35th, 65th, and 95th percentiles.

To evaluate the combined effects of DI-GM and genetic factors on GI cancer risk, participants were categorized into nine groups based on DI-GM and CPRS categories. We assessed both additive and multiplicative interaction models, as they provide complementary information about the joint effects of risk factors. Additive interaction measures whether the combined effect of two risk factors is greater than the sum of their individual effects (important for public health implications and prevention strategies), while multiplicative interaction assesses whether the combined effect exceeds the product of individual effects (important for understanding biological mechanisms). Using participants with low DI-GM and high genetic risk as the reference group, additive interaction was evaluated by calculating the Relative Excess Risk due to Interaction (RERI) and Attributable Proportion (AP) using the delta method to determine significance. Multiplicative interaction was measured using likelihood ratio tests.

In subgroup analyses, the models were reanalyzed stratified by age, sex, BMI, education level, the Townsend deprivation index, smoking status, alcohol consumption, physical activity, and family cancer history. We conducted a series of sensitivity analyses to assess the robustness of our primary findings: (1) reconstructing the DI-GM by substituting broader pulses intake for the chickpea component; (2) excluding cranberries due to UK Biobank not recording the specific type of dried fruit consumption [32]; (3) excluding participants diagnosed with GI cancer within the first two years of follow-up to minimize the risk of reverse causality; (4) excluding individuals who completed the 24-hour online dietary recall questionnaire only once, to ensure that the average intake of all dietary components accurately reflected habitual intake; and (5) excluding individuals with unknown or missing covariates. All statistical analyses were performed using R software version 4.3.1, with a two-tailed *P*-value of < 0.05 considered statistically significant.

## Result

### Baseline characteristics of participants

Among the 178,148 participants enrolled in the study, 2,682 individuals (1.51%) were diagnosed with GI cancer during a median follow-up period of 13.47 years (IQR: 12.87–14.27 years). Baseline characteristics classified according to the DI-GM are shown in Table 1 and Supplementary Table 5. Individuals ranked in the highest group of DI-GM tended to be older, female, exhibit lower BMI, possess higher education levels, have a lower Townsend deprivation index, never smoke, have no alcohol consumption and have higher physical activity. Furthermore, they reported higher consumption of avocado, broccoli, coffee, cranberries, fermented dairy, fiber, green tea, soybean, and whole grains.

**Table 1:** Baseline characteristics of 178,148 UK biobank participants across levels of DI-GM

Characteristics	DI-GM			Total (*N* = 178,148)
	Low (*N* = 67,109)	Moderate (*N* = 68,711)	High (*N* = 42,328)	
Age, year	55.03 ± 8.00	56.24 ± 7.86	57.03 ± 7.68	55.97 ± 7.91
BMI, kg/m^2^	27.59 ± 4.82	26.90 ± 4.51	25.93 ± 4.23	26.93 ± 4.61
Total energy intake, kJ/d	8,608.17 ± 2,406.20	8,636.34 ± 2,458.56	8,759.94 ± 2,212.25	8,655.09 ± 2,383.01
Townsend deprivation index	-1.58 ± 2.86	-1.75 ± 2.78	-1.73 ± 2.78	-1.68 ± 2.81
Sex				
Female	33,896 (50.51)	37,341 (54.35)	24,995 (59.05)	96,232 (54.02)
male	33,213 (49.49)	31,370 (45.65)	17,333 (40.95)	81,916 (45.98)
Annual household Income, Pound				
< 31,000	23,297 (34.72)	24,003 (34.93)	15,173 (35.85)	62,473 (35.01)
≥ 31,000	37,160 (55.37)	37,808 (55.02)	23,063 (54.49)	98,031 (55.03)
Unknown/missing	6,652 (9.91)	6,900 (10.05)	4,092 (9.66)	17,644 (9.96)
Education level				
No above	6,538 (9.74)	5,857 (8.52)	2,687 (6.35)	15,082 (8.47)
Medium	35,609 (53.06)	33,044 (48.09)	18,211 (43.02)	86,864 (48.76)
High	24,665 (36.75)	29,556 (43.01)	21,296 (50.31)	75,517 (42.39)
Unknown/missing	297 (0.45)	254 (0.47)	134 (0.32)	685 (0.38)
Smoking status				
Never	36,740 (54.75)	38,850 (56.54)	24,762 (58.50)	100,352 (56.33)
Previous	23,390 (34.85)	24,848 (36.16)	15,335 (36.23)	63,573 (35.65)
Current	6,837 (10.19)	4,855 (7.07)	2,160 (5.10)	13,852 (7.78)
Unknown/missing	142 (0.21)	158 (0.23)	71 (0.17)	371 (0.24)
Alcohol consumption				
Never	1,767 (2.63)	1,709 (2.49)	1,083 (2.56)	4,559 (2.56)
Previous	1,874 (2.79)	1,935 (2.82)	1,358 (3.21)	5,167 (2.90)
Current	63,433 (95.52)	65,046 (94.67)	39,873 (94.20)	168,352 (94.50)
Unknown/missing	35 (0.06)	21 (0.02)	14 (0.03)	70 (0.04)
Family history of cancer				
No	43,233 (64.42)	44,183 (64.30)	27,005 (63.80)	114,421 (64.23)
Yes	23,438 (34.93)	24,228 (35.26)	15,144 (35.78)	62,810 (35.26)
Unknown/missing	438 (0.65)	300 (0.44)	179 (0.42)	917 (0.51)
Physical activity level				
Low	11,848 (17.65)	9,830 (14.31)	4,677 (11.05)	26,355 (14.80)
Medium	40,774 (60.76)	42,446 (61.77)	26,115 (61.70)	109,335 (61.37)
High	14,487 (21.59)	16,435 (23.92)	11,536 (27.25)	42,458 (23.83)

Data are presented as mean ± (standard deviation) for continuous variables and n (%) for categorical variables

Low: DI-GM ≤ 4; moderate: 5 ≤ DI-GM ≤ 6; high: DI-GM ≥ 7

Abbreviations: BMI, body mass index; DI-GM, dietary index for gut microbiota

### Association of DI-GM with the risk of GI cancer

**Fig. 1:**
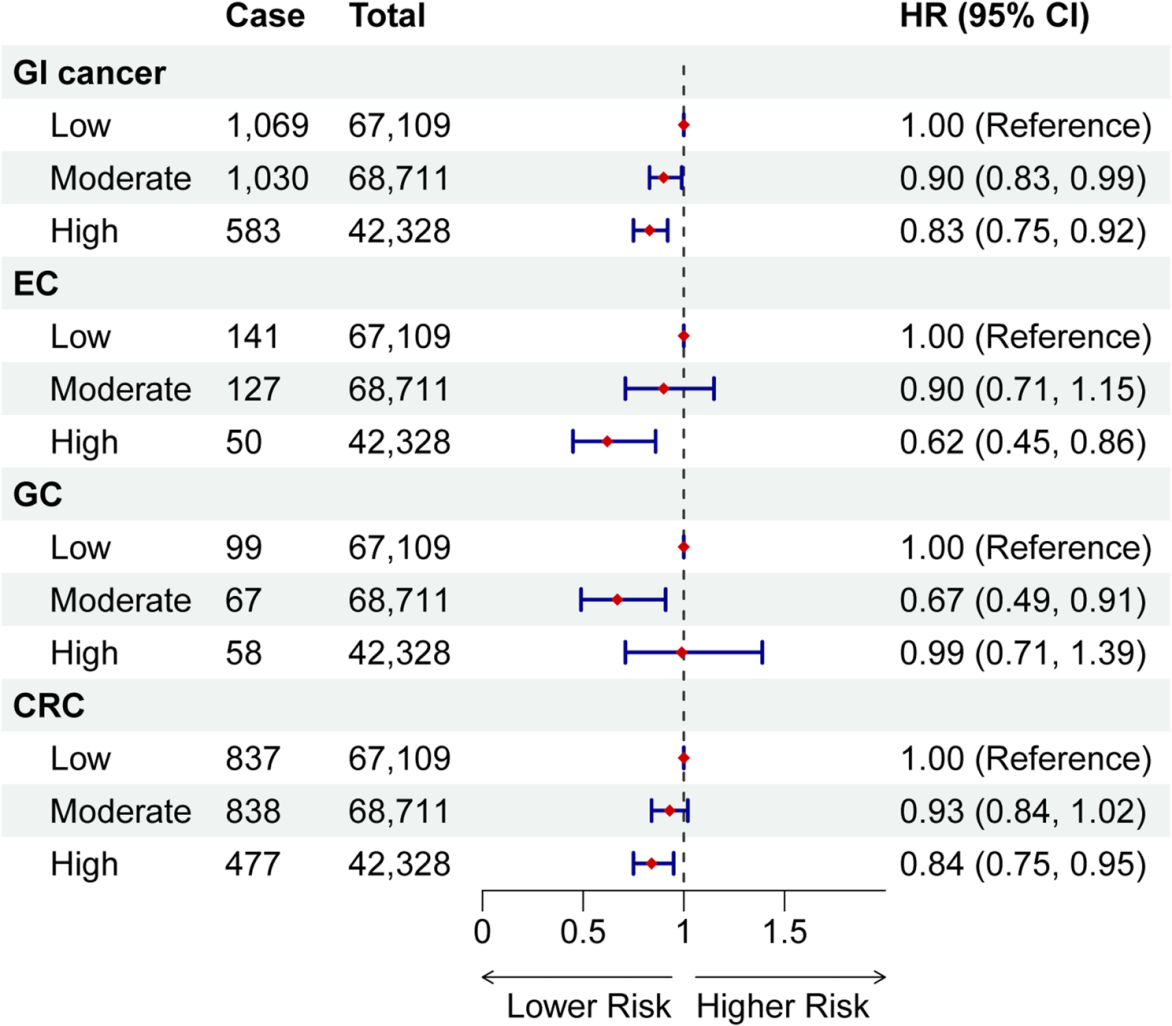
Associations of DI-GM with risk of gastrointestinal cancer. Low: DI-GM ≤ 4; moderate: 5 ≤ DI-GM ≤ 6; high: DI-GM ≥ 7. The models adjusted for age, sex, body mass index, total energy intake, annual household income, education level, Townsend deprivation index, smoking status, alcohol consumption, family history of cancer, and physical activity level. Abbreviations: CI, confidence interval; CRC, colorectal cancer; DI-GM: dietary index for gut microbiota; EC, esophageal cancer; GC, gastric cancer; GI: gastrointestinal; HR, hazard ratio

**Fig. 2:**
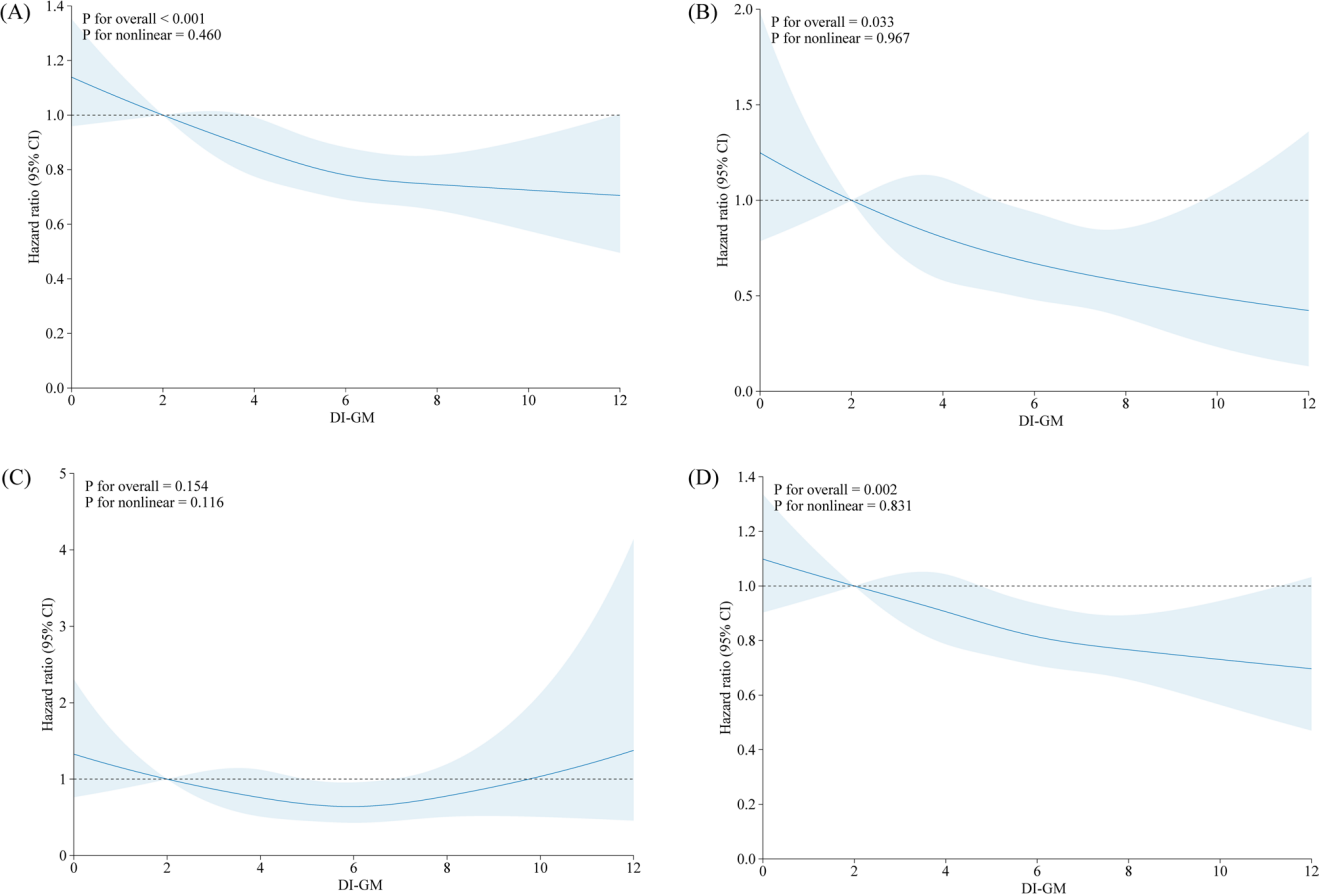
Restricted cubic splines for DI-GM and risk of (**A**) gastrointestinal cancer, (**B**) esophageal cancer, (**C**) gastric cancer, and (**D**) colorectal cancer. The models adjusted for age, sex, body mass index, total energy intake, annual household income, education level, Townsend deprivation index, smoking status, alcohol consumption, family history of cancer, and physical activity level. Abbreviations: CI, confidence interval; DI-GM: dietary index for gut microbiota

The DI-GM was significantly associated with a decreased risk of GI cancer in all models. In model 2, after adjustment for potential confounders, each additional SD of DI-GM was linked to a 5% decrease in GI cancer risk (HR: 0.95, 95% CI: 0.93–0.97; Supplementary Table 6). Participants with the highest DI-GM category had a 17% lower risk of GI cancer compared with those in the lowest DI-GM group (HR: 0.83, 95% CI: 0.75–0.92; *P* for trend < 0.001; Fig. 1). Additionally, restricted cubic spline analysis showed that the DI-GM associated, in a linear dose–response manner, with GI cancer risk (*P* for non-linearity > 0.05; Fig. 2). Further analysis of site-specific GI cancer revealed that each SD increase of DI-GM was associated with a 16% decreased risk of EC (95% CI: 0.75–0.94) and an 8% decreased risk of CRC (95% CI: 0.88–0.96). Compared with the lowest DI-GM group, the highest category was linked with a reduced risk of EC (HR: 0.62, 95% CI: 0.45–0.86), and CRC (HR: 0.84, 95% CI: 0.75–0.95), with all *P* for trend < 0.05. Linear association and dose–response manner were also observed (all *P* for non-linearity > 0.05; Fig. 2). For GC, association was significant in the middle DI-GM category (HR: 0.67, 95% CI: 0.49–0.91), but not in the highest group (HR: 0.99, 95% CI: 0.71–1.39; Supplementary Table 7). Analysis of individual food items showed that fermented dairy, fiber, whole grains, and meat had significant associations with GI cancer (all *P* < 0.05; Supplementary Table 8).

In subgroup analysis, we observed significant associations between DI-GM and GI cancer risk in participants who were older, male, overweight, non-smokers, drinkers, had high education level and low Townsend deprivation index, and maintained physical activity. Additionally, significant interactions were observed between DI-GM and age, sex, the Townsend deprivation index, and smoking status on GI cancer risk (*P* for interaction < 0.05; Supplementary Table 9). The significant associations between DI-GM and GI cancer risk remained robust after recalculating the DI-GM, excluding participants diagnosed with GI cancer during the first two years of follow-up, as well as those who completed the online 24-hour dietary recall questionnaire only once and had unknown or missing covariates (Supplementary Tables 10–14).

### Association of genetic risk with the risk of GI cancer

**Fig. 3:**
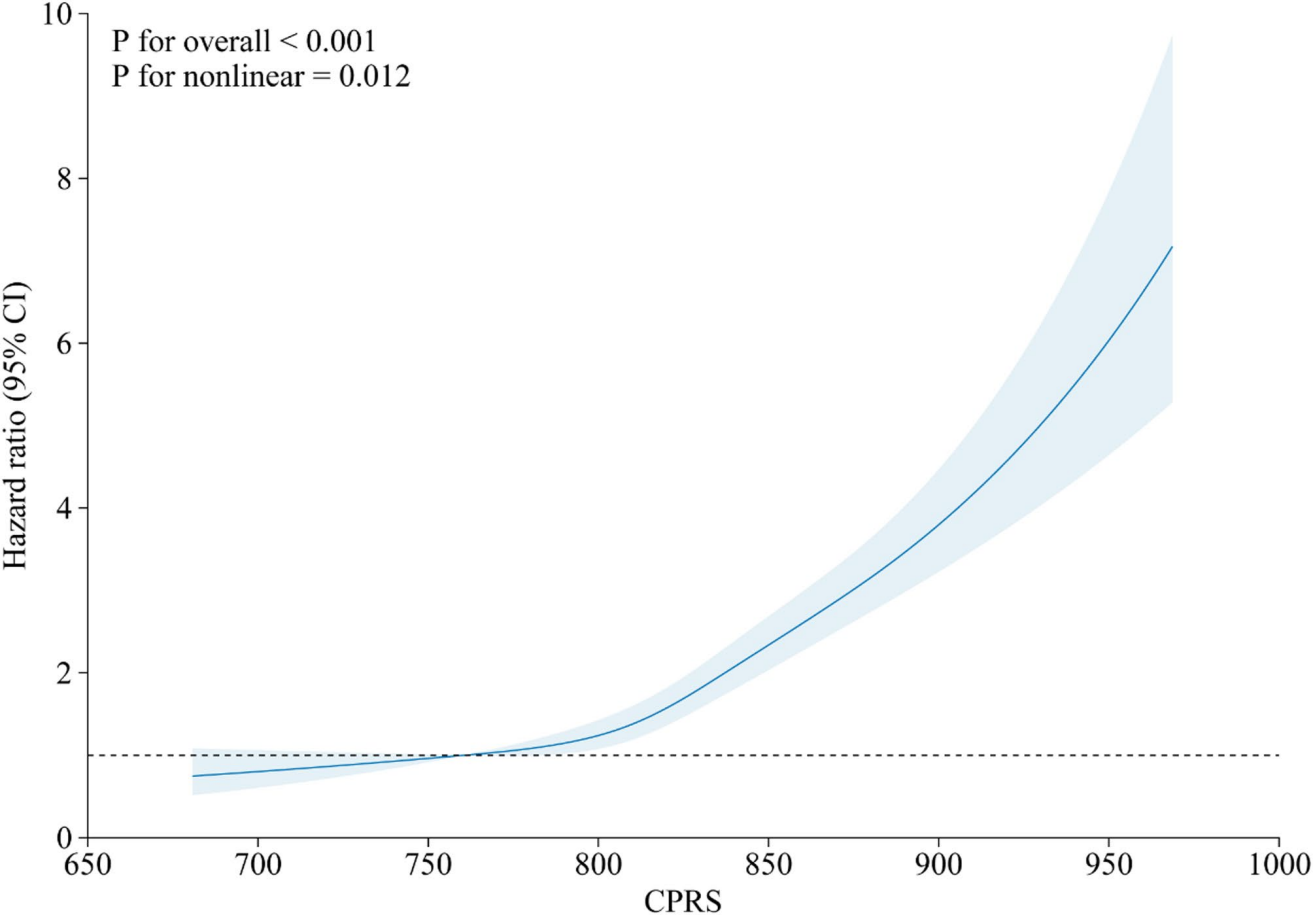
Restricted cubic splines for CPRS and risk of gastrointestinal cancer. The models adjusted for age, sex, body mass index, total energy intake, annual household income, education level, Townsend deprivation index, smoking status, alcohol consumption, family history of cancer, and physical activity level. Abbreviations: CI, confidence interval; CPRS, cancer polygenic risk score. Abbreviations: CI, confidence interval; HR, hazard ratio

In the fully adjusted model, a per SD increase in genetic risk was associated with a 48% higher risk of developing GI cancer (95% CI: 1.42–1.54; Supplementary Table 15). Compared with participants with low genetic risk, those with moderate and high genetic risk had a significantly higher risk of GI cancer, with HRs (95% CIs) of 1.65 (1.46–1.87) and 2.89 (2.53–3.29), respectively (Supplementary Table 15). Restricted cubic spline analysis showed a nonlinear association between genetic risk and GI cancer (*P* for non-linearity = 0.012; Fig. 3), which indicated a rapid increase in risk among populations with extremely high genetic risk.

### Joint association and interaction of DI-GM and genetic risk on GI cancer risk

**Fig. 4:**
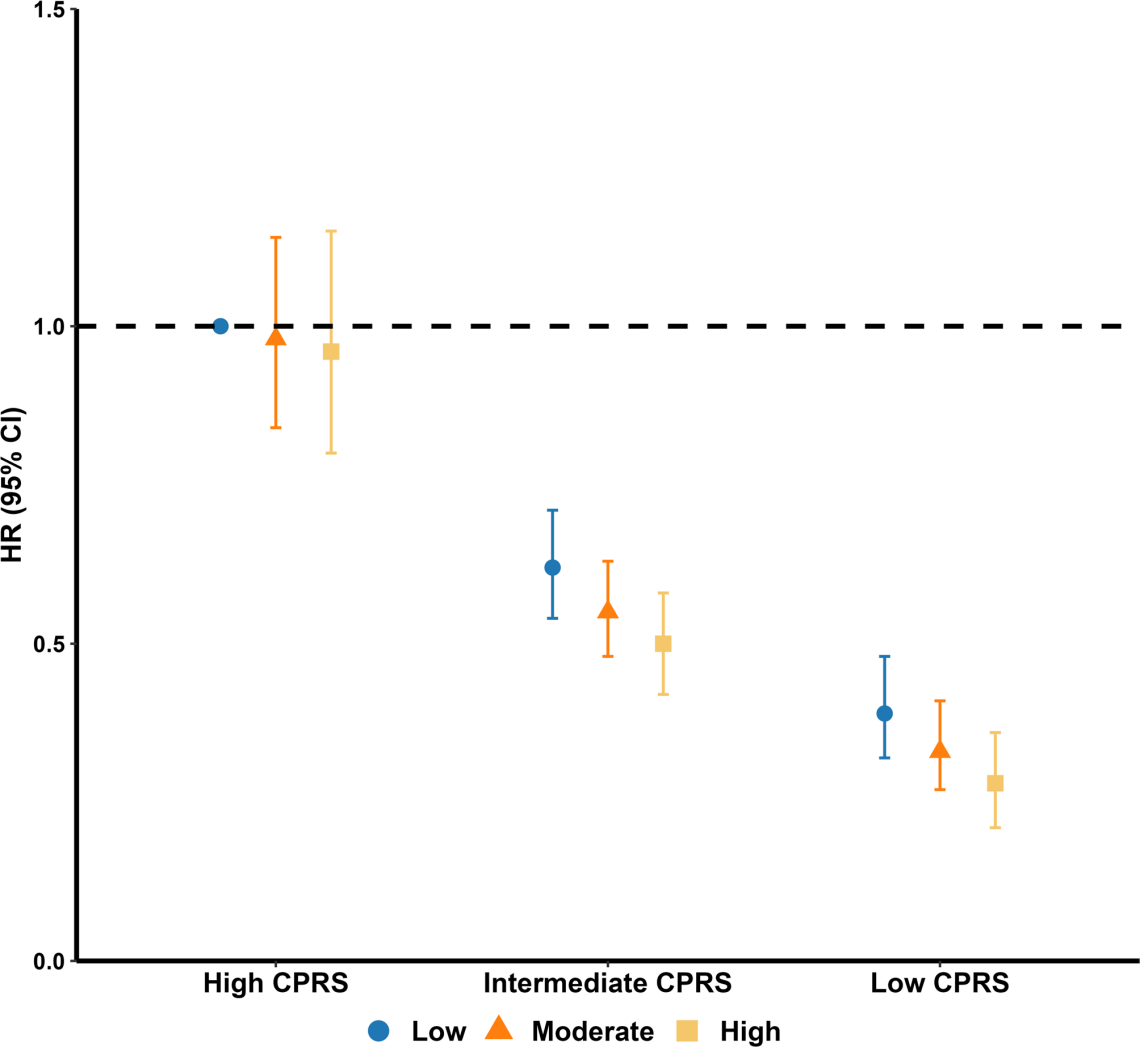
Joint effect of DI-GM and genetic risk on gastrointestinal cancer incidence. Low: DI-GM ≤ 4; moderate: 5 ≤ DI-GM ≤ 6; high: DI-GM ≥ 7. The models adjusted for age, sex, body mass index, energy intake, annual household income, education level, Townsend deprivation index, smoking status, alcohol consumption, family history of cancer, and physical activity level. Abbreviations: CI, confidence interval; CPRS, cancer polygenic risk score; HR, hazard ratio

Joint analysis showed that GI cancer risk decreased with both elevation of DI-GM and reduction of genetic risk in a dose-response manner. When compared with participants in the low DI-GM and high genetic risk group, those with high DI-GM and low genetic risk showed the lowest risk of GI cancer (HR: 0.28, 95% CI: 0.21–0.36; Fig. 4, Supplementary Table 16). Similar association was also observed in EC and CRC risk, with HR (95% CI) of 0.27 (0.11–0.63) and 0.21 (0.16–0.30), respectively (Supplementary Tables 17–18). Significant additive interactions between DI-GM and genetic risk on the risk of GI cancer were observed. Taking participants with low DI-GM and high genetic risk as a reference, the RERI (95% CI) for those with high DI-GM and low genetic risk was -0.09 (-0.16, -0.01) for the risk of GI cancer. This negative RERI value indicates that the protective effect of having both high DI-GM and low genetic risk is greater than would be expected from simply adding their individual protective effects. The AP value of 0.17 indicated that if both low DI-GM and high genetic risk did not exist, the incidence of GI cancer would decrease by approximately 17%. However, no significant multiplicative interaction was observed (Supplementary Table 19).

## Discussion

In this large-scale prospective cohort study based on the UK Biobank, we found a significant association between adherence to the high DI-GM diet and the reduced risk of GI cancers, with a significant linear dose-response relationship. Additionally, higher genetic risk, as quantified by the CPRS, was strongly associated with increased GI cancer risk. Importantly, we observed a significant additive interaction between DI-GM and genetic risk. These findings underscore the importance of considering both dietary factors that influence gut microbiota and genetic risk in GI cancer prevention strategies.

Our findings regarding the protective association of DI-GM with GI cancer risk align with previous research on dietary patterns and cancer risk, while providing novel insights specific to gut microbiota-related dietary components. Traditional dietary patterns such as the Healthy Eating diet, Mediterranean diet and healthful plant-based diet have been associated with reduced GI cancer risk [20, 33], whereas Western dietary patterns have shown positive associations with these cancers [34, 35]. However, the DI-GM offers a more targeted approach by specifically focusing on dietary components that influence gut microbiota composition and function, providing a potential mechanistic link between diet and cancer development.

The differential associations observed across GI cancer sites in our study are consistent with site-specific etiological factors identified in previous research, and may reflect differences in local microbiota composition and the varying influence of diet-microbiota interactions [6, 7]. The inconsistent association for gastric cancer, where a significant protective association was observed in the middle DI-GM category but not in the highest, warrants further discussion. This pattern may be primarily attributed to the dominant role of Helicobacter pylori infection in gastric carcinogenesis [36], which is estimated to account for 60–90% of gastric cancers worldwide. Unlike colorectal cancer, where dietary factors play a more central etiological role, the relative contribution of dietary factors to gastric cancer risk may be more limited or complex in the context of H. pylori infection. Additionally, gastric cancer is notably heterogeneous, with distinct subtypes (intestinal and diffuse) and anatomical locations (cardia and non-cardia) that may respond differently to dietary influences [37]. Our inability to stratify by these subtypes represents a limitation in fully characterizing the relationship between DI-GM and gastric cancer risk.

Our analysis of individual dietary components revealed significant associations between fermented dairy, fiber, whole grains, and meat consumption with GI cancer risk, corroborating previous findings. Meta-analyses have consistently shown inverse associations between dietary fiber and whole grains intake and colorectal cancer risk [38], while processed meat consumption has been classified as a Group 1 carcinogen for colorectal cancer by the International Agency for Research on Cancer [39]. The protective association of fermented dairy products observed in our study adds to the growing evidence suggesting beneficial effects of these foods on gut microbiota and cancer prevention [40, 41].

The DI-GM includes key dietary components that promote a healthy gut microbiome, such as dietary fiber, legumes, fermented dairy products, and whole grains [42–46], while limiting foods associated with gut imbalances, such as high-fat diets, red meat, and refined grains [12, 47, 48]. This approach is consistent with previous findings on how diet influences gut microbiota health, emphasizing dietary patterns that can either benefit or impair gut health. High DI-GM pattern helps maintain the health of gut microbiome and promotes the growth of beneficial bacteria, which intervene in cancer development through multiple pathways. For instance, soy foods can increase the levels of bifidobacteria and lactobacilli [49], while broccoli provides sulfur compounds that may prevent the excessive growth of certain sulfur metabolizing bacteria [50]. Sufficient dietary fiber intake boosts the production of short-chain fatty acids, particularly butyrate, which supports colonic mucosal barrier function and reduces inflammation [51]. Additionally, fermented dairy products rich in probiotics [52], along with polyphenol-rich foods like coffee, green tea, and cranberries, help inhibit the overgrowth of harmful bacteria [53–55]. These protective mechanisms likely contribute to the observed inverse relationship between higher DI-GM and the risk of GI cancer.

In addition to microbial factors, genetic risk plays a critical role in the development of GI cancer. Emerging evidence indicates that individuals with higher polygenic risk scores are more likely to undergo malignant transformation when exposed to adverse lifestyle factors [56, 57]. Our study confirmed that a higher GI-CPRS corresponds to an elevated risk of GI cancer, aligning with results from other large-scale studies [15, 58]. The additive interaction observed between DI-GM and genetic risk suggests that dietary factors may partially mitigate genetic risk to GI cancer. The observed interaction pattern is consistent with several previous gene-diet studies [59, 60]. From a mechanistic perspective, this pattern may reflect parallel biological pathways through which diet-influenced gut microbiota composition and genetic factors affect GI cancer risk. While genetic variants may influence cellular processes such as proliferation, DNA repair, and inflammation, dietary components may modify the gut microbiome in ways that partially counteract these genetic effects without necessarily operating through the same molecular pathways [61–63]. Additionally, certain dietary components such as phytonutrients may attenuate the effects of genetic variants by influencing pathways, DNA repair mechanisms, or inflammatory processes [64, 65].

Our findings have significant implications for clinical practice and public health strategies. The DI-GM offers a novel tool for assessing gut microbiota-related dietary patterns, with evidence suggesting even modest improvements could associate with reduced GI cancer risk through a linear dose-response relationship. The additive interaction between DI-GM and genetic risk highlights the value of personalized dietary interventions based on genetic risk stratification. Practical dietary recommendations can be derived from our findings on individual components: increasing consumption of fermented dairy, fiber-rich foods, and whole grains while limiting red and processed meat intake consistent with established guidelines for overall health [66]. Additionally, the varying associations across GI cancer sites suggest the need for targeted prevention strategies, particularly for EC and CRC, where dietary interventions appear more effective.

Our study has several key strengths. First, it is the first to use the UK Biobank to examine the relationship between the DI-GM, a dietary quality index associated with gut microbiota health, and the risk of GI cancer across different genetic risk levels. Second, the large sample size of the UK Biobank cohort provides sufficient statistical power, and the comprehensive data available allows for adjustment of a wide range of covariates, thereby enhancing the reliability of our findings. Additionally, the inclusion of genetic data allowed us to construct the specific PRS to predict GI cancer risk and incorporate genetic risk into our analyses. Third, the prospective study design and long median follow-up period enhance the temporal inference between DI-GM and GI cancer.

Despite these strengths, several limitations merit consideration. As an observational study, we cannot definitively establish causality, although the observed dose-response relationship supports a possible causal link. Dietary intake was self-reported, potentially introducing recall bias, but the use of repeated measures helps to mitigate this concern. Additionally, a notable limitation of our study is the incomplete adaptation of the DI-GM to the UK Biobank dietary data. Specifically, the original DI-GM developed by Kase et al. consists of 14 dietary components, but our adaptation includes only 13 components due to the absence of chickpea-specific intake data in the Oxford WebQ dietary assessment tool. To address this limitation, we conducted sensitivity analyses with results remaining consistent. Finally, the cohort primarily comprised White British individuals, potentially limiting the generalizability of our findings to other ethnicities. The DI-GM components were validated predominantly in Western populations, and their impact could differ in other ethnic groups with different baseline gut microbiota profiles. Future research should validate the DI-GM and its association with GI cancer risk in more ethnically diverse populations, potentially adapting the index to incorporate culturally relevant foods for different ethnic groups. This would improve the global applicability of microbiota-based dietary recommendations for cancer prevention.

## Conclusions

Higher DI-GM was associated with a lower risk of GI cancer, including EC and CRC. Individuals with both high DI-GM and low genetic risk had the lowest risk of GI cancer with a significant interaction. These findings suggest the importance of promoting a healthy gut microbiome through dietary interventions, and considering genetic risk in GI cancer prevention.

## Electronic supplementary material

Below is the link to the electronic supplementary material.

Supplementary Material 1

## Data Availability

Data used in this study are available on application to the UK Biobank (www.ukbiobank.ac.uk). Analytical methods and study materials will be available to other researchers from the corresponding authors upon reasonable request for the purposes of reproducing the results or replicating the procedure.

## Abbreviations

AP: Attributable Proportion
BMI: Body Mass Index
CI: Confidence Interval
CRC: Colorectal Cancer
CPRS: Cancer Polygenic Risk Scores
DI-GM: Dietary Index for Gut Microbiota
EC: Esophageal Cancer
GC: Gastric Cancer
HR: Hazard Ratio
IQR: Interquartile Range
RERI: Relative Excess Risk due to Interaction
SD: Standard Deviation
SNPs: Single Nucleotide Polymorphisms
