# Supplementary material for: Dietary index for gut microbiota and risk of gastrointestinal cancer: a prospective gene-diet study
Source: Nutr J. 2025 May 17;24:81. doi: 10.1186/s12937-025-01151-3 (PMC12085843; doi:10.1186/s12937-025-01151-3)
Supplement: Supplementary file 1 — Supplementary Material 1 [file 12937_2025_1151_MOESM1_ESM.docx]

**Supplementary materials**

**Dietary index for gut microbiota and risk of gastrointestinal cancer: a prospective gene-diet study**

Supplementary Figure 1. Flow chart of the study design

Supplementary Method. Construction of CPRS for GI cancer

Supplementary Table 1. Components of the DI-GM

Supplementary Table 2. Definition of portion size and food items used in this study

Supplementary Table 3. The Definition of variables in this study.

Supplementary Table 4. List of single-nucleotide polymorphisms used to build the polygenic risk scores of GI cancer

Supplementary Table 5. Baseline characteristics of food items of 178,148 UK Biobank participants across levels of DI-GM

Supplementary Table 6. Association between DI-GM and risk of gastrointestinal cancer

Supplementary Table 7. Association between DI-GM and risk of site-specific gastrointestinal cancer

Supplementary Table 8. Association between individual food items and risk of gastrointestinal cancer

Supplementary Table 9. Subgroup analysis for the association between DI-GM and risk of gastrointestinal cancer

Supplementary Table 10. Sensitivity analyses for excluding participants with less than two years of follow-up

Supplementary Table 11. Sensitivity analyses for recalculating the DI-GM after including pulses intake to reflect chickpea

Supplementary Table 12. Sensitivity analyses for recalculating the DI-GM after excluding cranberries

Supplementary Table 13. Sensitivity analysis for excluding participants who completed the questionnaire only once

Supplementary Table 14. Sensitivity analyses for excluding participants with unknown or missing covariates data

Supplementary Table 15. Association between genetic Risk and risk of gastrointestinal cancer

Supplementary Table 16. The combined effects of DI-GM and genetic risk with risk of gastrointestinal cancer

Supplementary Table 17. The combined effects of DI-GM and genetic risk with risk of esophageal cancer

Supplementary Table 18. The combined effects of DI-GM and genetic risk with risk of colorectal cancer

Supplementary Table 19. Additive and multiplicative interaction of DI-GM and genetic risk on gastrointestinal cancer

**
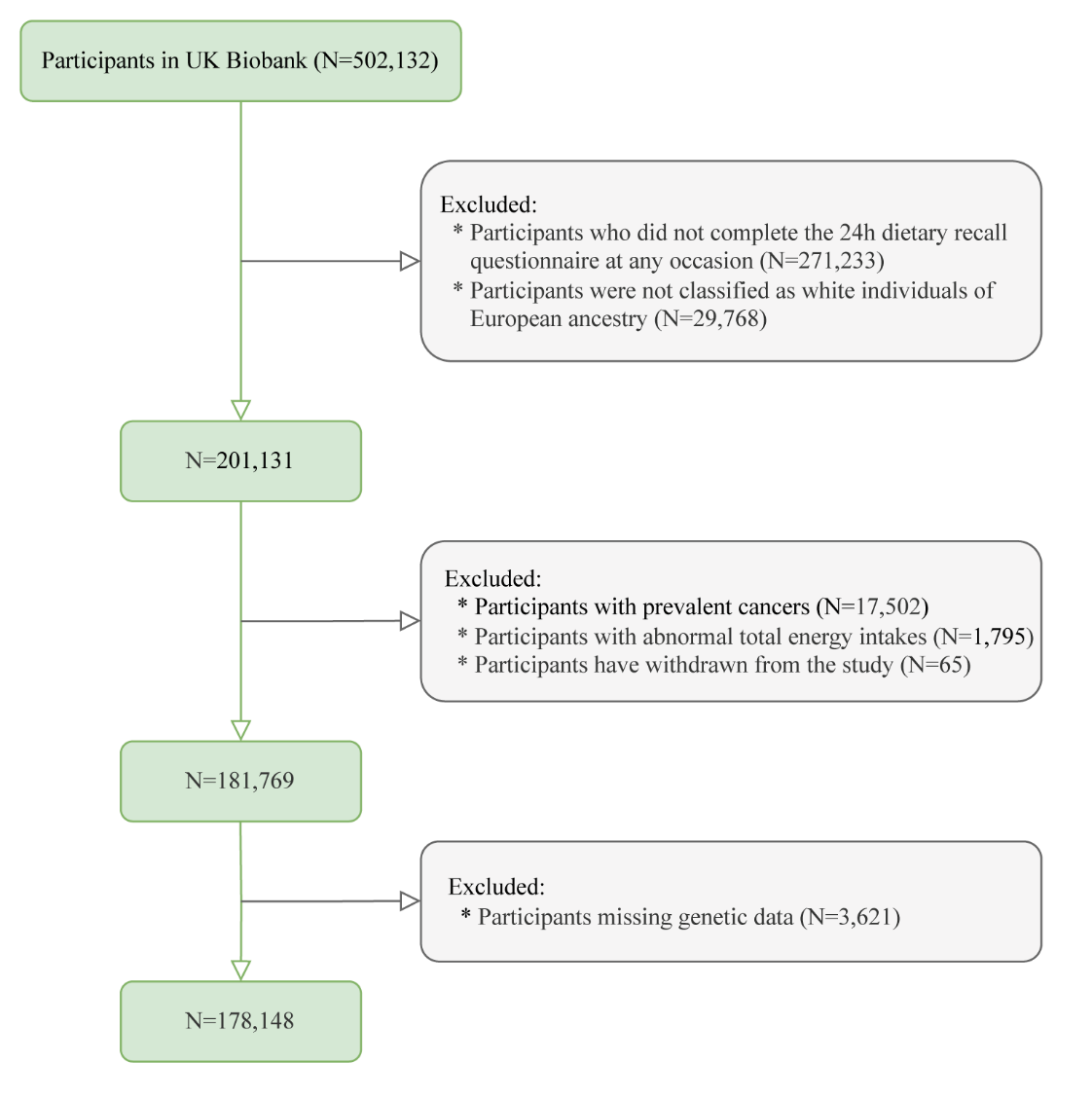
**

**Supplementary Figure 1. Flow chart of the study design**

**Supplementary Method. Construction of CPRS for GI cancer**

The procedure for genotyping and imputation of single-nucleotide polymorphisms (SNPs) used in the UK Biobank study has been described elsewhere in more detail.(1) We calculated cancer-specific PRSs based on independent SNPs for each cancer type from previously published GWAS. For each relevant GWAS, we reviewed the original manuscripts and supplementary materials to extract variants with a minor allele frequency (MAF) ≥ 0.01 and a P-value < 5×10⁻⁸. Variants were included in the analysis if they were either newly identified with a P-value < 5×10⁻⁸ or replicated with a consistent association direction and a P-value < 0.05 in the largest GWAS by sample size(2-6) (**Supplementary Table 4**). The corresponding effect sizes (i.e., odds ratios, ORs) were extracted from the largest studies. In cases where multiple correlated variants were reported for the same locus, we selected the SNPs with the smallest reported P-value using the linkage disequilibrium clumping procedure (at r² < 0.2) in PLINK. For variants not available in the UK Biobank genotyping data, we included strongly correlated SNPs (r² > 0.8) as proxies. SNPs with allele mismatches or MAF differences greater than 0.10 compared to the European population data from the 1000 Genomes Project were excluded.

To create a measure of genetic risk for overall cancer, we calculated the CPRS using the following method:

We constructed a CPRS to indicate the genetic risk of overall cancer:

$$\mathrm{CPRS}_{i}={h_{1}\times PRS}_{i,1}+h_{2}\times\mathrm{PRS}_{i,2}+\ldots+{h_{k}\times PRS}_{i,k}$$

where $\mathrm{CPRS}_{i}$ is the polygenic risk score for ith individual of overall cancer, PRS_i,k_ is the genetic risk score for cancer k, h_k_ is the age-standardized incidence of individual cancers in UK population (<https://www.ons.gov.uk/>). In this study, age-standardized incidence in men are 22.2, 14.4 and 81.9 for esophageal, gastric and colorectal cancer, respectively. For women, age-standardized incidence are 8.1, 6.2 and 54.9 for esophageal, gastric and colorectal cancer, respectively.

The main reasoning process was as follows:

We hypothesized that the PRS for overall cancer should be calculated as follows:

$$\mathrm{PRS}_{i}={\beta_{1}\times SNP}_{i,1,1}+\beta_{2}\times\mathrm{SNP}_{i,1,2}+\ldots+{\beta_{j}\times SNP}_{i,k,j}$$

where $\beta_{j}$ is per-allele log odds ratio (OR) for overall cancer associated with $\mathrm{SNP}_{k,j}$. We assumed that the genetic susceptibility loci of overall cancer were making up by those from each site-specific cancer, then $\mathrm{SNP}_{k,j}$ represented the susceptibility SNP j of site-specific cancer k. If $\beta_{k,j}$ represented the effect size of $\mathrm{SNP}_{k,j}$for site-specific cancer k, then the effect size of $\mathrm{SNP}_{k,j}$for overall cancer should be ${P_{k}\times\beta}_{k,j}$, where $P_{k}$ is the proportion of site-specific cancer k in overall cancer. The proportion of site-specific cancer k can be represented by$\frac{h_{k}}{h_{\mathrm{overall}}}$, where $h_{k}$ is the age-standardized incidence of site-specific cancer k in UK population and $h_{\mathrm{overall}}$ is the incidence of overall cancer. As a result, we can derive the following derivation:

$$\mathrm{PRS}_{i}={\frac{h_{1}}{h_{\mathrm{overall}}}\times\beta_{1,1}\times SNP}_{i,1,1}+\ldots+{\frac{h_{k}}{h_{\mathrm{overall}}}\times\beta_{k,j}\times SNP}_{i,k,j}$$

which can also be rewritten as:

$$\mathrm{PRS}_{i}={\frac{h_{1}}{h_{\mathrm{overall}}}\times PRS}_{i,1}+{\frac{h_{2}}{h_{\mathrm{overall}}}\times PRS}_{i,2}\ldots+{\frac{h_{k}}{h_{\mathrm{overall}}}\times PRS}_{i,k}$$

and then:

$${h_{\mathrm{overall}}\times PRS}_{i}={h_{k}\times PRS}_{i,1}+{h_{k}\times PRS}_{i,2}\ldots+{h_{k}\times PRS}_{i,k}$$

Because ${h_{\mathrm{overall}}\times PRS}_{i}$ has the same effect in risk stratification of population as that of $\mathrm{PRS}_{i}$, we used $\mathrm{CPRS}_{i}$ (equivalent to ${h_{\mathrm{overall}}\times PRS}_{i}$) to represent the genetic risk score of overall cancer in the present study. According to the CPRS quintile classification, low risk corresponds to Q1, intermediate risk to Q2-Q4, and high risk to Q5(7).

**Supplementary Table 1. Components of the DI-GM**

| **Component^1^** | **Foods Component** | **Scoring** |
| --- | --- | --- |
| **Beneficial to gut microbiota** | |  |
| Avocados | Avocados | For each component, a score of 1 if consumption at or above the sex-specific median, else 0 |
| Broccoli | Broccoli |  |
| Chickpea | Chickpeas |  |
| Coffee | Coffee |  |
| Cranberries | Cranberries |  |
| Fermented dairy | Yogurt, cheese, kefir, sour cream, buttermilk |  |
| Fiber | Fiber |  |
| Green tea | Green tea |  |
| Soybean | Soy products—Soy milk, Tofu |  |
| Whole grains | Grains defined as whole grains, containing the entire grain kernel—the bran, germ, and endosperm |  |
| **Unfavorable to gut microbiota** | |  |
| High-fat diet  (% energy) | Not applicable | 0 if consumption at or above 40% energy from fat, else 1 |
| Processed meat | Frankfurters, sausages, corned beef, and luncheon meat made from beef, pork, or poultry | For each remaining component, a score of 0 if consumption at or above the sex-specific median, else 1 |
| Red meat | Beef, veal, pork, lamb, and game meat; excludes organ meat and cured meat |  |
| Refined grains | Refined grains that do not contain all components of the entire grain kernel |  |

^1^ Dietary Index for Gut Microbiota (DI-GM) is constructed by Kase et al. (8).

**Supplementary Table 2.** **Definition of portion size and food items used in this study**

| **Component** | **Value Type** | **Food items based on the Oxford WebQ questionnaire** |
| --- | --- | --- |
| Avocados | Categorical, 1 portion: 136 g | Avocado |
| Broccoli | Categorical, 1 portion: 80 g | Broccoli |
| Chickpea ^1^ | Not applicable | Not applicable |
| Coffee | Continuous, g | Coffee |
| Cranberries ^2^ | Continuous, g | Dried fruit |
| Fermented dairy | Continuous, g | Yogurt; Cheese |
| Fiber | Continuous, g | Englyst fiber |
| Green tea | Categorical, 1 portion: 190 g | Green tea |
| Soybean | Continuous, g  Categorical, 1 portion: 90/125g | Soy milk; Tofu (1 portion: 90g); Soya dessert (1 portion: 125g) |
| Whole grains | Continuous, g | Wholemeal bread; Wholemeal pasta, brown rice and other wholegrains; Muesli; Bran cereal; Other cereal; Oat cereal |
| High-fat diet | Continuous, KJ | Fat; Energy |
| Processed meat | Continuous, g | Processed meat |
| Red meat | Continuous, g | Beef; Pork; Lamb |
| Refined grains | Continuous, g | White bread; Other bread; Biscuit cereal; Biscuits; Pizza; Other desserts and cakes and pastries; Savoury snacks; Samosa, pakora; Savoury crackers; Grain dishes |

^1^ Chickpeas unavailable due to UK Biobank not recording the consumption.

^2^ Cranberries are substituted using dried fruit, which includes cranberries.

**Supplementary Table 3. The Definition of variables in this study.**

| **Variables** | **Field IDs** | **Definitions of variables** |
| --- | --- | --- |
| **Outcomes** | | |
| Cancer prevalence | 40006 | ICD 10 code. |
|  | 40013 | ICD 9 code. |
|  | 20001 | Data-Coding in the UK biobank. |
| Cancer incidence | 40006 | ICD 10 code. |
| Lost follow-up | 191 | Indicates the date from which a person is believed to be lost to follow-up. |
| **Exposures ^1^** | | |
| Avocado intake | 104100 | "How many medium avocados did you have?" |
| Broccoli intake | 104140 | "How many servings of broccoli did you have?" |
| Pulses intake | 104010 | How many servings of other beans (kidney beans/chick peas/butter beans etc) or lentils did you have? |
| Coffee, caffeinated | 26081 | Estimated intake from past 24 hours. |
| Coffee, decaffeinated | 26082 | Estimated intake from past 24 hours. |
| Dried fruit ^2^ | 26092 | Estimated intake from past 24 hours. |
| Full fat yogurt | 26096 | Estimated intake from past 24 hours. |
| Low fat yogurt | 26102 | Estimated intake from past 24 hours. |
| High fat cheese | 26099 | Estimated intake from past 24 hours. |
| Medium and  low fat cheese | 26103 | Estimated intake from past 24 hours. |
| Englyst fiber | 26017 | Estimated intake from past 24 hours. |
| Green tea | 100420 | "How many cups/mugs of green tea did you drink yesterday?" |
| Soy milk | 26136 | Estimated intake from past 24 hours. |
| Tofu intake | 103270 | "How many servings of tofu/tempeh/TVP/soya mince did you have?" |
| Soya dessert intake | 102170 | "How many servings/individual pots of soya ice cream, soya yogurt, other soya dessert did you have?" |
| Wholemeal bread | 26074 | Estimated intake from past 24 hours. |
| Wholemeal pasta, brown rice and  other wholegrains | 26114 | Estimated intake from past 24 hours. |
| Muesli | 26105 | Estimated intake from past 24 hours. |
| Bran cereal | 26076 | Estimated intake from past 24 hours. |
| Other cereal | 26079 | Estimated intake from past 24 hours. |
| Oat cereal (sugar) | 26078 | Estimated intake from past 24 hours. |
| Oat cereal | 26077 | Estimated intake from past 24 hours. |
| Fat | 26008 | Fat from overall diet. Estimated intake from past 24 hours. |
| Energy | 26002 | Energy from overall diet. Estimated intake from past 24 hours. |
| Processed meat | 26122 | Estimated intake from past 24 hours. |
| Beef | 26066 | Estimated intake from past 24 hours. |
| Pork | 26117 | Estimated intake from past 24 hours. |
| Lamb | 26100 | Estimated intake from past 24 hours. |
| White bread | 26073 | Estimated intake from past 24 hours. |
| Other bread | 26072 | Estimated intake from past 24 hours. |
| Biscuit cereal | 26075 | Estimated intake from past 24 hours. |
| Biscuits | 26068 | Estimated intake from past 24 hours. |
| Pizza | 26116 | Estimated intake from past 24 hours. |
| Other desserts and cakes and pastries | 26085 | Estimated intake from past 24 hours. |
| Savoury snacks | 26134 | Estimated intake from past 24 hours. |
| Samosa, pakora | 26128 | Estimated intake from past 24 hours. |
| Savoury crackers | 26083 | Estimated intake from past 24 hours. |
| Grain dishes | 26097 | Estimated intake from past 24 hours. |
| **Covariates** | | |
| Age at recruitment | 21022 | Age was treated as a continuous variable. |
| Sex | 31 | Data-Coding in the UK biobank: male; female. |
| Annual household income | 738 | Data-Coding in the UK biobank: “(Less than £18,000)”, “(£18,000 to £30,999)”, “(£31,000 to £51,999)”, “(£52,000 to £100,000)”, “(Greater than £100,000)”, “Do not know”, “Prefer not to answer”. We combined “(Less than £18,000)” and “(£18,000 to £30,999)” into a group, “(£31,000 to £51,999)”, “(£52,000 to £100,000)”, and “(Greater than £100,000)” into a group and created an “unknown/missing” group response category for “Do not know”, “Prefer not to answer”, or missing data. |
| Education levels | 6138 | Data-Coding in the UK biobank: “College or university degree”, “A level / AS levels or equivalent”, “O levels / GCSEs or equivalent”, “CSEs or equivalent”, “NVQ or HND or HNC or equivalent”, “Other professional qualifications”, “None of the above”, “Prefer not to answer”. We combined “College or university degree” into a group, “A level / AS levels or equivalent” and “O levels / GCSEs or equivalent” and “CSEs or equivalent” and “NVQ or HND or HNC or equivalent” and “Other professional qualifications” into a group, “None of the above” into a group, and created an “unknown/missing” response category for “Prefer not to answer” or missing data. |
| Townsend deprivation index | 22189 | Townsend deprivation index calculated immediately prior to participant joining UK Biobank. Based on the preceding national census output areas. Each participant is assigned a score corresponding to the output area in which their postcode is located. Townsend deprivation index was treated as a continuous variable and missing value with the average fill. |
| Body mass index | 21001 | Body mass index was treated as a continuous variable. |
| Smoking status | 20116 | Data-Coding in the UK biobank: “Never”, “Previous”, “Current”, “Prefer not to answer”. We created an “unknown/missing” response category for “Prefer not to answer” or missing data. |
| Alcohol status | 20117 | Data-Coding in the UK biobank: “Never”, “Previous”, “Current”, “Prefer not to answer”. We created an “unknown/missing” response category for “Prefer not to answer” or missing data. |
| Physical activity | 22040 | Total Metabolic Equivalent Task minutes per week for all activity including walking, moderate and vigorous activity. |
| Family history of cancer | 20112 | Having family history of cancer was defined that any of relatives had cancer history. |
|  | 20113 |  |
|  | 20114 |  |
|  | 20107 |  |
|  | 20110 |  |
|  | 20111 |  |

^1^ Total weight of corresponding food group. Estimated intake from past 24 hours (9, 10). Whole grains and Refined grains specific food items refer to relevant literature (11, 12).

^2^ Cranberries are substituted using dried fruit, which includes cranberries.

**Supplementary Table 4. List of single-nucleotide polymorphisms used to build the polygenic risk scores of GI cancer**

| **Index** | **ICD10-code** | **Cancer site** | **SNP rsID ^1^** | **CHR** | **Position (GRCh37)** | **Risk Allele** | **Other Allele** | **Weight** | **Study (PMID)** | **Included** |
| --- | --- | --- | --- | --- | --- | --- | --- | --- | --- | --- |
| 1 | C15 | Esophagus | rs7255 | 2 | 20878820 | T | C | 0.153064376 | 27527254 | Included |
| 2 | C15 | Esophagus | rs9823696 | 3 | 183783353 | A | G | 0.158284979 | 27527254 | Included |
| 3 | C15 | Esophagus | rs7428643 | 5 | 668309 | C | T | 0.285404481 | 27527254 | Included |
| 4 | C15 | Esophagus | rs62416609 | 6 | 62391538 | A | C | 0.190785635 | 27527254 | Included |
| 5 | C15 | Esophagus | rs2188554 | 7 | 117040117 | A | G | 0.20880119 | 27527254 | Included |
| 6 | C16 | Stomach | rs2990223 | 1 | 155184975 | G | T | 0.235722334 | 32588423 | Included |
| 7 | C16 | Stomach | rs10036575 | 5 | 40685795 | T | C | 0.210721031 | 32588423 | Included |
| 8 | C16 | Stomach | rs2294008 | 8 | 143761931 | T | C | 0.19062036 | 32588423 | Included |
| 9 | C18-C20 | Colon, Rectum | rs2807367 | 1 | 22503282 | G | C | 0.038 | 36539618 | Included |
| 10 | C18-C20 | Colon, Rectum | rs34963268 | 1 | 22710877 | G | C | 0.066 | 36539618 | Included |
| 11 | C18-C20 | Colon, Rectum | rs61776719 | 1 | 38461319 | C | A | 0.056 | 36539618 | Included |
| 12 | C18-C20 | Colon, Rectum | rs3124454 | 1 | 71040166 | G | T | 0.041 | 36539618 | Included |
| 13 | C18-C20 | Colon, Rectum | rs5028523 | 1 | 173000000 | A | G | 0.041 | 36539618 | Included |
| 14 | C18-C20 | Colon, Rectum | rs8179460 | 1 | 183056222 | T | C | 0.071 | 36539618 | Included |
| 15 | C18-C20 | Colon, Rectum | rs12137232 | 1 | 202000000 | G | T | 0.039 | 36539618 | Included |
| 16 | C18-C20 | Colon, Rectum | rs12078075 | 1 | 205000000 | G | A | 0.071 | 36539618 | Included |
| 17 | C18-C20 | Colon, Rectum | rs201395236 | 1 | 245181421 | T | C | 0.528 | 36539618 | Removed after LD clumping |
| 18 | C18-C20 | Colon, Rectum | rs7606562 | 2 | 48686695 | T | A | 0.02 | 36539618 | Included |
| 19 | C18-C20 | Colon, Rectum | rs11692435 | 2 | 98275354 | G | A | 0.091 | 36539618 | Included |
| 20 | C18-C20 | Colon, Rectum | rs3731861 | 2 | 219191256 | T | C | 0.056 | 36539618 | Included |
| 21 | C18-C20 | Colon, Rectum | rs2001732 | 3 | 52880740 | C | T | 0.074 | 36539618 | Included |
| 22 | C18-C20 | Colon, Rectum | rs2581817 | 3 | 53071797 | G | C | 0.047 | 36539618 | Included |
| 23 | C18-C20 | Colon, Rectum | rs7623129 | 3 | 64624426 | C | T | 0.037 | 36539618 | Included |
| 24 | C18-C20 | Colon, Rectum | rs13086367 | 3 | 112903888 | A | G | 0.054 | 36539618 | Removed after LD clumping |
| 25 | C18-C20 | Colon, Rectum | rs12635946 | 3 | 112916918 | C | T | 0.056 | 36539618 | Included |
| 26 | C18-C20 | Colon, Rectum | rs72942485 | 3 | 112999560 | G | A | 0.146 | 36539618 | Included |
| 27 | C18-C20 | Colon, Rectum | rs113569514 | 3 | 133748789 | T | C | 0.08 | 36539618 | Included |
| 28 | C18-C20 | Colon, Rectum | rs10936599 | 3 | 169492101 | C | T | 0.053 | 36539618 | Included |
| 29 | C18-C20 | Colon, Rectum | rs1426947 | 4 | 175000000 | T | C | 0.041 | 36539618 | Included |
| 30 | C18-C20 | Colon, Rectum | rs55810369 | 5 | 40090784 | C | T | 0.052 | 36539618 | Included |
| 31 | C18-C20 | Colon, Rectum | rs3930345 | 5 | 82881255 | C | T | 0.045 | 36539618 | Included |
| 32 | C18-C20 | Colon, Rectum | rs12659017 | 5 | 125988175 | G | A | 0.018 | 36539618 | Included |
| 33 | C18-C20 | Colon, Rectum | rs1294437 | 6 | 6749789 | C | T | 0.04 | 36539618 | Included |
| 34 | C18-C20 | Colon, Rectum | rs9379084 | 6 | 7231843 | G | A | 0.07 | 36539618 | Included |
| 35 | C18-C20 | Colon, Rectum | rs2516452 | 6 | 31427095 | A | G | 0.073 | 36539618 | Included |
| 36 | C18-C20 | Colon, Rectum | rs16878812 | 6 | 35569562 | A | G | 0.09 | 36539618 | Included |
| 37 | C18-C20 | Colon, Rectum | rs62404966 | 6 | 55712124 | C | T | 0.067 | 36539618 | Included |
| 38 | C18-C20 | Colon, Rectum | rs6912214 | 6 | 55721302 | T | C | 0.044 | 36539618 | Included |
| 39 | C18-C20 | Colon, Rectum | rs6928864 | 6 | 105966894 | C | A | 0.093 | 36539618 | Included |
| 40 | C18-C20 | Colon, Rectum | rs1182197 | 7 | 2863289 | A | C | 0.039 | 36539618 | Included |
| 41 | C18-C20 | Colon, Rectum | rs7810512 | 7 | 45150331 | A | C | 0.05 | 36539618 | Included |
| 42 | C18-C20 | Colon, Rectum | rs12539962 | 7 | 73167259 | C | T | 0.039 | 36539618 | Included |
| 43 | C18-C20 | Colon, Rectum | rs2527927 | 7 | 99477426 | G | A | 0.041 | 36539618 | Included |
| 44 | C18-C20 | Colon, Rectum | rs17686932 | 7 | 130600748 | A | G | 0.117 | 36539618 | Included |
| 45 | C18-C20 | Colon, Rectum | rs60911071 | 8 | 23664632 | G | C | 0.062 | 36539618 | Included |
| 46 | C18-C20 | Colon, Rectum | rs2450115 | 8 | 117624093 | T | C | 0.085 | 36539618 | Included |
| 47 | C18-C20 | Colon, Rectum | rs6983267 | 8 | 128413305 | G | T | 0.151 | 36539618 | Included |
| 48 | C18-C20 | Colon, Rectum | rs7013278 | 8 | 128414892 | T | C | 0.143 | 36539618 | Removed after LD clumping |
| 49 | C18-C20 | Colon, Rectum | rs4733767 | 8 | 128581578 | G | A | 0.05 | 36539618 | Included |
| 50 | C18-C20 | Colon, Rectum | rs7859362 | 9 | 22105927 | T | C | 0.055 | 36539618 | Included |
| 51 | C18-C20 | Colon, Rectum | rs34405347 | 9 | 101679752 | T | G | 0.06 | 36539618 | Included |
| 52 | C18-C20 | Colon, Rectum | rs10978941 | 9 | 110000000 | C | T | 0.061 | 36539618 | Included |
| 53 | C18-C20 | Colon, Rectum | rs7038489 | 9 | 137000000 | C | T | 0.074 | 36539618 | Included |
| 54 | C18-C20 | Colon, Rectum | rs11255841 | 10 | 8739580 | T | A | 0.111 | 36539618 | Included |
| 55 | C18-C20 | Colon, Rectum | rs1775910 | 10 | 29096942 | G | C | 0.04 | 36539618 | Included |
| 56 | C18-C20 | Colon, Rectum | rs10821905 | 10 | 52646093 | G | A | 0.063 | 36539618 | Included |
| 57 | C18-C20 | Colon, Rectum | rs4919687 | 10 | 104595248 | G | A | 0.028 | 36539618 | Included |
| 58 | C18-C20 | Colon, Rectum | rs174537 | 11 | 61552680 | G | T | 0.064 | 36539618 | Included |
| 59 | C18-C20 | Colon, Rectum | rs10751097 | 11 | 69938433 | A | G | 0.046 | 36539618 | Included |
| 60 | C18-C20 | Colon, Rectum | rs117042741 | 11 | 74628743 | C | T | 0.226 | 36539618 | Included |
| 61 | C18-C20 | Colon, Rectum | rs55864876 | 11 | 100717136 | G | A | 0.068 | 36539618 | Included |
| 62 | C18-C20 | Colon, Rectum | rs2155065 | 11 | 101616011 | C | T | 0.038 | 36539618 | Included |
| 63 | C18-C20 | Colon, Rectum | rs3087967 | 11 | 111156836 | T | C | 0.111 | 36539618 | Included |
| 64 | C18-C20 | Colon, Rectum | rs10774214 | 12 | 4368352 | T | C | 0.045 | 36539618 | Included |
| 65 | C18-C20 | Colon, Rectum | rs7398375 | 12 | 57540848 | C | G | 0.068 | 36539618 | Included |
| 66 | C18-C20 | Colon, Rectum | rs7297628 | 12 | 64404555 | T | C | 0.038 | 36539618 | Removed after LD clumping |
| 67 | C18-C20 | Colon, Rectum | rs11178634 | 12 | 71518329 | G | T | 0.045 | 36539618 | Included |
| 68 | C18-C20 | Colon, Rectum | rs11108175 | 12 | 96050887 | A | G | 0.055 | 36539618 | Included |
| 69 | C18-C20 | Colon, Rectum | rs10161980 | 13 | 34093518 | C | G | 0.049 | 36539618 | Included |
| 70 | C18-C20 | Colon, Rectum | rs45597035 | 13 | 73649152 | A | G | 0.055 | 36539618 | Included |
| 71 | C18-C20 | Colon, Rectum | rs1078563 | 13 | 110000000 | G | C | 0.041 | 36539618 | Included |
| 72 | C18-C20 | Colon, Rectum | rs4901473 | 14 | 54445157 | G | A | 0.044 | 36539618 | Included |
| 73 | C18-C20 | Colon, Rectum | rs17094983 | 14 | 59189361 | G | A | 0.064 | 36539618 | Included |
| 74 | C18-C20 | Colon, Rectum | rs61975764 | 14 | 93014929 | G | A | 0.025 | 36539618 | Included |
| 75 | C18-C20 | Colon, Rectum | rs1554865 | 15 | 32999806 | C | T | 0.121 | 36539618 | Included |
| 76 | C18-C20 | Colon, Rectum | rs3809570 | 15 | 67000117 | C | A | 0.054 | 36539618 | Included |
| 77 | C18-C20 | Colon, Rectum | rs9924886 | 16 | 68743939 | A | C | 0.056 | 36539618 | Included |
| 78 | C18-C20 | Colon, Rectum | rs3936188 | 16 | 80036840 | A | G | 0.066 | 36539618 | Included |
| 79 | C18-C20 | Colon, Rectum | rs7206216 | 16 | 86338288 | A | T | 0.045 | 36539618 | Included |
| 80 | C18-C20 | Colon, Rectum | rs4968127 | 17 | 809643 | G | A | 0.062 | 36539618 | Included |
| 81 | C18-C20 | Colon, Rectum | rs1791373 | 18 | 3616779 | T | A | 0.041 | 36539618 | Included |
| 82 | C18-C20 | Colon, Rectum | rs2337113 | 18 | 46452327 | A | G | 0.172 | 36539618 | Included |
| 83 | C18-C20 | Colon, Rectum | rs62131228 | 19 | 1157642 | G | A | 0.108 | 36539618 | Included |
| 84 | C18-C20 | Colon, Rectum | rs28840750 | 19 | 33519927 | T | G | 0.193 | 36539618 | Included |
| 85 | C18-C20 | Colon, Rectum | rs966816 | 20 | 6376481 | G | A | 0.095 | 36539618 | Included |
| 86 | C18-C20 | Colon, Rectum | rs994308 | 20 | 6603622 | C | T | 0.069 | 36539618 | Included |
| 87 | C18-C20 | Colon, Rectum | rs6059938 | 20 | 33187130 | G | A | 0.035 | 36539618 | Included |
| 88 | C18-C20 | Colon, Rectum | rs6066825 | 20 | 47340117 | A | G | 0.081 | 36539618 | Included |
| 89 | C18-C20 | Colon, Rectum | rs6012915 | 20 | 48986567 | C | T | 0.06 | 36539618 | Included |
| 90 | C18-C20 | Colon, Rectum | rs6095946 | 20 | 49060191 | C | T | 0.065 | 36539618 | Included |
| 91 | C18-C20 | Colon, Rectum | rs6014965 | 20 | 55831203 | A | G | 0.047 | 36539618 | Included |
| 92 | C18-C20 | Colon, Rectum | rs2738783 | 20 | 62308612 | T | G | 0.053 | 36539618 | Included |
| 93 | C18-C20 | Colon, Rectum | rs4616575 | 22 | 29406076 | T | G | 0.041 | 36539618 | Included |
| 94 | C18-C20 | Colon, Rectum | rs130651 | 22 | 39644273 | G | A | 0.049 | 36539618 | Included |
| 95 | C18-C20 | Colon, Rectum | rs7542665 | 1 | 62673037 | C | T | 0.019 | 36539618 | Included |
| 96 | C18-C20 | Colon, Rectum | rs6660031 | 1 | 110365045 | A | G | 0.04 | 36539618 | Included |
| 97 | C18-C20 | Colon, Rectum | rs6691170 | 1 | 222045446 | T | G | 0.057 | 36539618 | Included |
| 98 | C18-C20 | Colon, Rectum | rs2078095 | 1 | 240000000 | G | A | 0.04 | 36539618 | Included |
| 99 | C18-C20 | Colon, Rectum | rs1446585 | 2 | 136407479 | G | A | 0.039 | 36539618 | Included |
| 100 | C18-C20 | Colon, Rectum | rs448513 | 2 | 159964552 | C | T | 0.044 | 36539618 | Included |
| 101 | C18-C20 | Colon, Rectum | rs4668039 | 2 | 169000000 | G | A | 0.042 | 36539618 | Included |
| 102 | C18-C20 | Colon, Rectum | rs6434979 | 2 | 199570718 | A | G | 0.048 | 36539618 | Included |
| 103 | C18-C20 | Colon, Rectum | rs4675253 | 2 | 199888532 | G | C | 0.062 | 36539618 | Included |
| 104 | C18-C20 | Colon, Rectum | rs1800734 | 3 | 37034946 | A | G | 0.024 | 36539618 | Included |
| 105 | C18-C20 | Colon, Rectum | rs35470271 | 3 | 40915239 | G | A | 0.081 | 36539618 | Included |
| 106 | C18-C20 | Colon, Rectum | rs704417 | 3 | 64252424 | T | C | 0.045 | 36539618 | Included |
| 107 | C18-C20 | Colon, Rectum | rs67550176 | 3 | 66444330 | C | T | 0.049 | 36539618 | Included |
| 108 | C18-C20 | Colon, Rectum | rs10049390 | 3 | 133701119 | A | G | 0.054 | 36539618 | Included |
| 109 | C18-C20 | Colon, Rectum | rs280097 | 4 | 94937860 | C | T | 0.046 | 36539618 | Included |
| 110 | C18-C20 | Colon, Rectum | rs2007403 | 4 | 106131210 | T | C | 0.052 | 36539618 | Included |
| 111 | C18-C20 | Colon, Rectum | rs2388976 | 4 | 116000000 | A | G | 0.037 | 36539618 | Included |
| 112 | C18-C20 | Colon, Rectum | rs11727676 | 4 | 145659064 | C | T | 0.093 | 36539618 | Included |
| 113 | C18-C20 | Colon, Rectum | rs10006803 | 4 | 152000000 | C | G | 0.036 | 36539618 | Included |
| 114 | C18-C20 | Colon, Rectum | rs77776598 | 5 | 1240998 | C | T | 0.14 | 36539618 | Included |
| 115 | C18-C20 | Colon, Rectum | rs2735940 | 5 | 1296486 | G | A | 0.082 | 36539618 | Included |
| 116 | C18-C20 | Colon, Rectum | rs1445012 | 5 | 40282106 | C | G | 0.096 | 36539618 | Included |
| 117 | C18-C20 | Colon, Rectum | rs647161 | 5 | 134499092 | A | C | 0.059 | 36539618 | Included |
| 118 | C18-C20 | Colon, Rectum | rs2302274 | 5 | 149546426 | G | A | 0.05 | 36539618 | Included |
| 119 | C18-C20 | Colon, Rectum | rs472959 | 5 | 172000000 | A | G | 0.038 | 36539618 | Included |
| 120 | C18-C20 | Colon, Rectum | rs2070699 | 6 | 12292772 | T | G | 0.052 | 36539618 | Included |
| 121 | C18-C20 | Colon, Rectum | rs209142 | 6 | 28862617 | C | G | 0.038 | 36539618 | Included |
| 122 | C18-C20 | Colon, Rectum | rs1476570 | 6 | 29809860 | A | G | 0.012 | 36539618 | Included |
| 123 | C18-C20 | Colon, Rectum | rs116353863 | 6 | 31010185 | C | T | 0.165 | 36539618 | Included |
| 124 | C18-C20 | Colon, Rectum | rs2517448 | 6 | 31062667 | C | T | 0.046 | 36539618 | Included |
| 125 | C18-C20 | Colon, Rectum | rs62401893 | 6 | 31317361 | A | G | 0.104 | 36539618 | Included |
| 126 | C18-C20 | Colon, Rectum | rs3830041 | 6 | 32191339 | T | C | 0.039 | 36539618 | Included |
| 127 | C18-C20 | Colon, Rectum | rs9271363 | 6 | 32586732 | C | G | 0.084 | 36539618 | Included |
| 128 | C18-C20 | Colon, Rectum | rs9470361 | 6 | 36623379 | A | G | 0.068 | 36539618 | Included |
| 129 | C18-C20 | Colon, Rectum | rs4711689 | 6 | 41692812 | A | G | 0.028 | 36539618 | Included |
| 130 | C18-C20 | Colon, Rectum | rs57939401 | 6 | 45572071 | A | G | 0.064 | 36539618 | Included |
| 131 | C18-C20 | Colon, Rectum | rs2208603 | 6 | 55577214 | C | T | 0.049 | 36539618 | Included |
| 132 | C18-C20 | Colon, Rectum | rs145997965 | 6 | 106000000 | C | T | 0.189 | 36539618 | Included |
| 133 | C18-C20 | Colon, Rectum | rs6911915 | 6 | 118000000 | C | T | 0.045 | 36539618 | Included |
| 134 | C18-C20 | Colon, Rectum | rs151127921 | 6 | 134000000 | T | C | 0.159 | 36539618 | Included |
| 135 | C18-C20 | Colon, Rectum | rs80077929 | 7 | 46094089 | T | C | 0.06 | 36539618 | Included |
| 136 | C18-C20 | Colon, Rectum | rs4236382 | 7 | 46889411 | A | G | 0.047 | 36539618 | Included |
| 137 | C18-C20 | Colon, Rectum | rs6948177 | 7 | 47510741 | G | A | 0.061 | 36539618 | Included |
| 138 | C18-C20 | Colon, Rectum | rs826732 | 8 | 59742639 | C | G | 0.041 | 36539618 | Included |
| 139 | C18-C20 | Colon, Rectum | rs16892766 | 8 | 117630683 | C | A | 0.202 | 36539618 | Included |
| 140 | C18-C20 | Colon, Rectum | rs117079142 | 8 | 117790914 | A | C | 0.18 | 36539618 | Included |
| 141 | C18-C20 | Colon, Rectum | rs11557154 | 9 | 34107505 | T | C | 0.052 | 36539618 | Included |
| 142 | C18-C20 | Colon, Rectum | rs10817106 | 9 | 113654654 | C | G | 0.048 | 36539618 | Included |
| 143 | C18-C20 | Colon, Rectum | rs11789898 | 9 | 137000000 | T | G | 0.053 | 36539618 | Included |
| 144 | C18-C20 | Colon, Rectum | rs12217641 | 10 | 8663875 | T | C | 0.007 | 36539618 | Included |
| 145 | C18-C20 | Colon, Rectum | rs1773860 | 10 | 29291556 | T | C | 0.038 | 36539618 | Included |
| 146 | C18-C20 | Colon, Rectum | rs704017 | 10 | 80819132 | G | A | 0.086 | 36539618 | Included |
| 147 | C18-C20 | Colon, Rectum | rs1782645 | 10 | 81048611 | T | C | 0.039 | 36539618 | Included |
| 148 | C18-C20 | Colon, Rectum | rs7071258 | 10 | 91574624 | A | G | 0.042 | 36539618 | Included |
| 149 | C18-C20 | Colon, Rectum | rs6584283 | 10 | 101290301 | C | T | 0.037 | 36539618 | Included |
| 150 | C18-C20 | Colon, Rectum | rs35564340 | 10 | 101344263 | A | G | 0.09 | 36539618 | Included |
| 151 | C18-C20 | Colon, Rectum | rs12241008 | 10 | 114280702 | C | T | 0.103 | 36539618 | Included |
| 152 | C18-C20 | Colon, Rectum | rs11196172 | 10 | 114726843 | A | G | 0.08 | 36539618 | Included |
| 153 | C18-C20 | Colon, Rectum | rs4444073 | 11 | 10331664 | C | A | 0.038 | 36539618 | Included |
| 154 | C18-C20 | Colon, Rectum | rs11236187 | 11 | 74364566 | C | A | 0.075 | 36539618 | Included |
| 155 | C18-C20 | Colon, Rectum | rs7946853 | 11 | 74409077 | C | T | 0.032 | 36539618 | Included |
| 156 | C18-C20 | Colon, Rectum | rs497916 | 11 | 119000000 | T | C | 0.041 | 36539618 | Included |
| 157 | C18-C20 | Colon, Rectum | rs3217810 | 12 | 4388271 | T | C | 0.123 | 36539618 | Included |
| 158 | C18-C20 | Colon, Rectum | rs3217874 | 12 | 4400808 | T | C | 0.067 | 36539618 | Included |
| 159 | C18-C20 | Colon, Rectum | rs10849432 | 12 | 6385727 | T | C | 0.062 | 36539618 | Included |
| 160 | C18-C20 | Colon, Rectum | rs10849434 | 12 | 6407044 | C | A | 0.062 | 36539618 | Included |
| 161 | C18-C20 | Colon, Rectum | rs77969132 | 12 | 31594813 | T | C | 0.307 | 36539618 | Removed after LD clumping |
| 162 | C18-C20 | Colon, Rectum | rs2730985 | 12 | 43130624 | G | A | 0.053 | 36539618 | Included |
| 163 | C18-C20 | Colon, Rectum | rs11169572 | 12 | 51216890 | C | T | 0.077 | 36539618 | Included |
| 164 | C18-C20 | Colon, Rectum | rs653178 | 12 | 112007756 | T | C | 0.076 | 36539618 | Included |
| 165 | C18-C20 | Colon, Rectum | rs9634162 | 12 | 115098094 | A | G | 0.043 | 36539618 | Included |
| 166 | C18-C20 | Colon, Rectum | rs7300312 | 12 | 115890922 | C | T | 0.061 | 36539618 | Included |
| 167 | C18-C20 | Colon, Rectum | rs7299936 | 12 | 116000000 | A | G | 0.052 | 36539618 | Removed after LD clumping |
| 168 | C18-C20 | Colon, Rectum | rs73208120 | 12 | 117747590 | G | T | 0.055 | 36539618 | Included |
| 169 | C18-C20 | Colon, Rectum | rs116964464 | 13 | 27543193 | T | C | 0.108 | 36539618 | Included |
| 170 | C18-C20 | Colon, Rectum | rs12427846 | 13 | 37491650 | C | T | 0.078 | 36539618 | Included |
| 171 | C18-C20 | Colon, Rectum | rs78341008 | 13 | 73791554 | C | T | 0.107 | 36539618 | Included |
| 172 | C18-C20 | Colon, Rectum | rs1886450 | 13 | 73986628 | G | A | 0.048 | 36539618 | Included |
| 173 | C18-C20 | Colon, Rectum | rs9318511 | 13 | 78601413 | C | A | 0.059 | 36539618 | Included |
| 174 | C18-C20 | Colon, Rectum | rs4600332 | 13 | 111078872 | G | A | 0.044 | 36539618 | Included |
| 175 | C18-C20 | Colon, Rectum | rs28611105 | 14 | 51359658 | G | T | 0.048 | 36539618 | Included |
| 176 | C18-C20 | Colon, Rectum | rs1497077 | 14 | 52491655 | C | T | 0.041 | 36539618 | Included |
| 177 | C18-C20 | Colon, Rectum | rs1951864 | 14 | 54369299 | A | G | 0.028 | 36539618 | Included |
| 178 | C18-C20 | Colon, Rectum | rs35107139 | 14 | 54419106 | C | A | 0.084 | 36539618 | Included |
| 179 | C18-C20 | Colon, Rectum | rs8020436 | 14 | 59208437 | A | G | 0.06 | 36539618 | Included |
| 180 | C18-C20 | Colon, Rectum | rs80158569 | 14 | 99782937 | A | G | 0.08 | 36539618 | Included |
| 181 | C18-C20 | Colon, Rectum | rs1919364 | 15 | 33009574 | G | C | 0.074 | 36539618 | Included |
| 182 | C18-C20 | Colon, Rectum | rs17816465 | 15 | 33156386 | A | G | 0.082 | 36539618 | Included |
| 183 | C18-C20 | Colon, Rectum | rs56324967 | 15 | 67402824 | C | T | 0.053 | 36539618 | Included |
| 184 | C18-C20 | Colon, Rectum | rs77148098 | 15 | 68075698 | A | G | 0.066 | 36539618 | Included |
| 185 | C18-C20 | Colon, Rectum | rs8031386 | 15 | 72508799 | A | C | 0.042 | 36539618 | Included |
| 186 | C18-C20 | Colon, Rectum | rs71407320 | 15 | 91185291 | T | C | 0.073 | 36539618 | Included |
| 187 | C18-C20 | Colon, Rectum | rs847208 | 16 | 86254051 | A | C | 0.032 | 36539618 | Included |
| 188 | C18-C20 | Colon, Rectum | rs62042090 | 16 | 86703949 | T | C | 0.055 | 36539618 | Included |
| 189 | C18-C20 | Colon, Rectum | rs12603526 | 17 | 800593 | C | T | 0.049 | 36539618 | Included |
| 190 | C18-C20 | Colon, Rectum | rs11247566 | 17 | 835371 | G | A | 0.038 | 36539618 | Included |
| 191 | C18-C20 | Colon, Rectum | rs1078643 | 17 | 10707241 | A | G | 0.087 | 36539618 | Included |
| 192 | C18-C20 | Colon, Rectum | rs983318 | 17 | 70413253 | A | G | 0.057 | 36539618 | Included |
| 193 | C18-C20 | Colon, Rectum | rs35204860 | 17 | 81053330 | C | T | 0.078 | 36539618 | Included |
| 194 | C18-C20 | Colon, Rectum | rs10409772 | 19 | 5840926 | A | C | 0.067 | 36539618 | Included |
| 195 | C18-C20 | Colon, Rectum | rs34797592 | 19 | 16417198 | T | C | 0.09 | 36539618 | Included |
| 196 | C18-C20 | Colon, Rectum | rs1800469 | 19 | 41860296 | G | A | 0.053 | 36539618 | Included |
| 197 | C18-C20 | Colon, Rectum | rs12979278 | 19 | 49218602 | T | C | 0.036 | 36539618 | Included |
| 198 | C18-C20 | Colon, Rectum | rs11670192 | 19 | 59017640 | A | G | 0.06 | 36539618 | Included |
| 199 | C18-C20 | Colon, Rectum | rs4813802 | 20 | 6699595 | G | T | 0.078 | 36539618 | Included |
| 200 | C18-C20 | Colon, Rectum | rs28488 | 20 | 6762221 | T | C | 0.05 | 36539618 | Included |
| 201 | C18-C20 | Colon, Rectum | rs2423279 | 20 | 7812350 | C | T | 0.073 | 36539618 | Included |
| 202 | C18-C20 | Colon, Rectum | rs6065668 | 20 | 42532821 | T | C | 0.035 | 36539618 | Included |
| 203 | C18-C20 | Colon, Rectum | rs6017248 | 20 | 42658274 | C | A | 0.049 | 36539618 | Included |
| 204 | C18-C20 | Colon, Rectum | rs6091189 | 20 | 49256285 | T | C | 0.04 | 36539618 | Included |
| 205 | C18-C20 | Colon, Rectum | rs13831 | 20 | 57475191 | G | A | 0.022 | 36539618 | Included |
| 206 | C18-C20 | Colon, Rectum | rs1741640 | 20 | 60932414 | C | T | 0.133 | 36539618 | Included |
| 207 | C18-C20 | Colon, Rectum | rs9983528 | 21 | 47772439 | A | G | 0.064 | 36539618 | Included |
| 208 | C18-C20 | Colon, Rectum | rs5751474 | 22 | 43689542 | A | G | 0.051 | 36539618 | Included |
| 209 | C18-C20 | Colon, Rectum | rs34256596 | 22 | 43778431 | A | G | 0.04 | 36539618 | Included |
| 210 | C18-C20 | Colon, Rectum | rs9614460 | 22 | 45745229 | G | T | 0.04 | 36539618 | Included |
| 211 | C18-C20 | Colon, Rectum | rs9330814 | 22 | 46364191 | T | C | 0.049 | 36539618 | Included |
| 212 | C18-C20 | Colon, Rectum | rs2732875 | X | 9763898 | C | G | 0.169 | 36539618 | Removed after LD clumping |

^1^ Single Nucleotide Polymorphisms (SNPs) were selected from previously published GWASs with the largest sample size for various subtypes of digestive system cancers.

Abbreviations: CHR, Chromosome; GIC, gastrointestinal cancer; SNP, single nucleotide polymorphism.

**Supplementary Table 5. Baseline characteristics of food items of 178,148 UK Biobank participants across levels of DI-GM**

| **Characteristics ^1^** | **DI-GM** | | | **Total**  **(N=178,148)** | ***P* value** |
| --- | --- | --- | --- | --- | --- |
|  | **Low**  **(N=67,109)** | **Moderate**  **(N=68,711)** | **High**  **(N=42,328)** |  |  |
| Avocado intake | 1.55 (12.46) | 4.15 (20.01) | 11.51 (31.89) | 4.92 (21.66) | < 0.001 |
| Broccoli intake | 14.90 (42.21) | 31.47 (58.02) | 58.76 (69.56) | 31.71 (58.31) | < 0.001 |
| Coffee intake | 246.70 (280.34) | 323.79 (287.73) | 357.83 (273.71) | 302.84 (285.33) | < 0.001 |
| Dried fruit intake | 1.67 (8.81) | 6.27 (17.93) | 17.28 (29.20) | 7.15 (19.79) | < 0.001 |
| Fermented dairy intake | 36.65 (49.08) | 65.83 (64.01) | 92.03 (66.91) | 61.06 (63.35) | < 0.001 |
| Fiber intake | 14.60 (5.11) | 18.24 (6.09) | 22.29 (6.39) | 17.83 (6.52) | < 0.001 |
| Green tea intake | 9.57 (68.58) | 24.73 (105.15) | 66.63 (161.61) | 28.97 (112.81) | < 0.001 |
| Soybean intake | 1.95 (19.74) | 7.16 (39.02) | 25.46 (70.21) | 9.55 (44.60) | < 0.001 |
| Whole grains intake | 46.74 (61.10) | 97.38 (85.95) | 143.34 (94.09) | 89.22 (88.09) | < 0.001 |
| Fat intake | 2,811.57 (1,056.42) | 2,666.97 (1,045.06) | 2,616.86 (948.40) | 2,709.54 (1,030.54) | < 0.001 |
| Processed meat intake | 26.74 (35.19) | 16.12 (27.69) | 8.65 (19.71) | 18.35 (30.09) | < 0.001 |
| Red meat intake | 52.40 (56.39) | 35.03 (49.39) | 20.11 (38.91) | 38.03 (51.54) | < 0.001 |
| Refined grains intake | 152.33 (98.81) | 115.02 (95.40) | 85.13 (73.22) | 121.97 (95.66) | < 0.001 |

Data are presented as mean (standard deviation).

Low: DI-GM ≤ 4; moderate: 5 ≤ DI-GM ≤ 6; high: DI-GM ≥ 7.

Abbreviations: DI-GM, dietary index for gut microbiota.

^1^ All food items are measured in g/day.

**Supplementary Table 6. Association between DI-GM and risk of gastrointestinal cancer**

| **DI-GM** | **Cases/Total** | **HR (95%CI)** | | |
| --- | --- | --- | --- | --- |
|  |  | **Crude model** | **Model 1 ^1^** | **Model 2 ^2^** |
| Low | 1,069/67,109 | 1.00 (Ref.) | 1.00 (Ref.) | 1.00 (Ref.) |
| Moderate | 1,030/68,711 | 0.94 (0.86, 1.02) | 0.90 (0.82, 0.98) | 0.90 (0.83, 0.99) |
| High | 583/42,328 | 0.86 (0.78, 0.95) | 0.82 (0.74, 0.91) | 0.83 (0.75, 0.92) |
| *P* for trend |  | 0.004 | <0.001 | <0.001 |
| Per SD increment | | 0.96 (0.94, 0.98) | 0.95 (0.93, 0.97) | 0.95 (0.93, 0.97) |

Low: DI-GM ≤ 4; moderate: 5 ≤ DI-GM ≤ 6; high: DI-GM ≥ 7.

Abbreviations: CI, confidence interval; DI-GM, dietary index for gut microbiota; HR, hazard ratio; Ref, reference; SD, standard deviation.

^1^ Model 1 was adjusted for age (continuous, year), sex (female or male), body mass index (continuous, kg/m^2^), and total energy intake (continuous, kJ/d).

^2^ Model 2 was further adjusted for annual household income (≥ 3100 pound or ≤ 3100 pound), education level (high, medium, or no above), Townsend deprivation index (continuous), smoking status (never, previous, or current), alcohol consumption (never, previous, or current), family history of cancer (no or yes), and physical activity level (high, medium, or low), based on model 1.

**Supplementary Table 7. Association between DI-GM and risk of site-specific gastrointestinal** **cancer**

| **DI-GM** | **Cases/Total** | **HR (95%CI)** | | |
| --- | --- | --- | --- | --- |
|  |  | **Crude model** | **Model 1 ^1^** | **Model 2 ^2^** |
| EC | | | | |
| Low | 141/67,109 | 1.00 (Ref.) | 1.00 (Ref.) | 1.00 (Ref.) |
| Moderate | 127/68,711 | 0.88 (0.69, 1.12) | 0.86 (0.68, 1.10) | 0.90 (0.71, 1.15) |
| High | 50/42,328 | 0.56 (0.41, 0.77) | 0.58 (0.42, 0.80) | 0.62 (0.45, 0.86) |
| *P* for trend |  | <0.001 | 0.001 | 0.007 |
| Per SD increment |  | 0.81 (0.72, 0.91) | 0.82 (0.73, 0.92) | 0.84 (0.75, 0.94) |
| GC | | | | |
| Low | 99/67,109 | 1.00 (Ref.) | 1.00 (Ref.) | 1.00 (Ref.) |
| Moderate | 67/68,711 | 0.66 (0.48, 0.90) | 0.64 (0.47, 0.88) | 0.67 (0.49, 0.91) |
| High | 58/42,328 | 0.93 (0.67, 1.28) | 0.93 (0.67, 1.29) | 0.99 (0.71, 1.39) |
| *P* for trend |  | 0.383 | 0.384 | 0.624 |
| Per SD increment |  | 0.91 (0.80, 1.04) | 0.91 (0.79, 1.04) | 0.93 (0.81, 1.07) |
| CRC | | | | |
| Low | 837/67,109 | 1.00 (Ref.) | 1.00 (Ref.) | 1.00 (Ref.) |
| Moderate | 838/68,711 | 0.98 (0.89, 1.07) | 0.93 (0.84, 1.02) | 0.93 (0.84, 1.02) |
| High | 477/42,328 | 0.90 (0.80, 1.01) | 0.84 (0.75, 0.94) | 0.84 (0.75, 0.95) |
| *P* for trend |  | 0.079 | 0.003 | 0.003 |
| Per SD increment |  | 0.95 (0.91, 0.99) | 0.92 (0.88, 0.96) | 0.92 (0.88, 0.96) |

Low: DI-GM ≤ 4; moderate: 5 ≤ DI-GM ≤ 6; high: DI-GM ≥ 7.

Abbreviations: CI, confidence interval; CRC, colorectal cancer; DI-GM, dietary index for gut microbiota; EC, esophageal cancer; GC, gastric cancer; HR, hazard ratio; Ref, reference; SD, standard deviation.

^1^ Model 1 was adjusted for age (continuous, year), sex (female or male), body mass index (continuous, kg/m^2^), and total energy intake (continuous, kJ/d).

^2^ Model 2 was further adjusted for annual household income (≥ 3100 pound or < 3100 pound), education level (high, medium, or no above), Townsend deprivation index (continuous), smoking status (never, previous, or current), alcohol consumption (never, previous, or current), family history of cancer (no or yes), and physical activity level (high, medium, or low), based on model 1.

**Supplementary Table 8. Association between individual food items and risk of gastrointestinal** **cancer**

| **Food item** | **Cases/Total** | **HR (95% CI)** | | |
| --- | --- | --- | --- | --- |
|  |  | **Crude model** | **Model 1 ^1^** | **Model 2 ^2^** |
| Avocado |  |  |  |  |
| < median | 2,532/167,150 | 1.00 (Ref.) | 1.00 (Ref.) | 1.00 (Ref.) |
| ≥ median | 150/10,998 | 0.90 (0.76, 1.06) | 0.97 (0.82, 1.15) | 0.96 (0.82, 1.14) |
| Broccoli |  |  |  |  |
| < median | 1,915/129,934 | 1.00 (Ref.) | 1.00 (Ref.) | 1.00 (Ref.) |
| ≥ median | 767/48,214 | 1.08 (0.99, 1.17) | 1.03 (0.95, 1.12) | 1.04 (0.96, 1.13) |
| Coffee |  |  |  |  |
| < median | 1,334/89,206 | 1.00 (Ref.) | 1.00 (Ref.) | 1.00 (Ref.) |
| ≥ median | 1348/88,942 | 1.01 (0.94, 1.09) | 0.95 (0.88, 1.03) | 0.94 (0.87, 1.02) |
| Cranberries |  |  |  |  |
| < median | 2,163/141,144 | 1.00 (Ref.) | 1.00 (Ref.) | 1.00 (Ref.) |
| ≥ median | 519/37,004 | 0.91 (0.83, 1.00) | 0.90 (0.82, 1.00) | 0.92 (0.83, 1.01) |
| Fermented dairy |  |  |  |  |
| < median | 1,402/89,320 | 1.00 (Ref.) | 1.00 (Ref.) | 1.00 (Ref.) |
| ≥ median | 1,280/88,828 | 0.92 (0.85, 0.99) | 0.87 (0.81, 0.94) | 0.88 (0.82, 0.95) |
| Fiber |  |  |  |  |
| < median | 1,354/89,074 | 1.00 (Ref.) | 1.00 (Ref.) | 1.00 (Ref.) |
| ≥ median | 1,328/89,074 | 0.98 (0.91, 1.06) | 0.88 (0.81, 0.96) | 0.90 (0.83, 0.98) |
| Green tea |  |  |  |  |
| < median | 2,481/162,792 | 1.00 (Ref.) | 1.00 (Ref.) | 1.00 (Ref.) |
| ≥ median | 201/15,356 | 0.86 (0.74, 0.99) | 0.92 (0.79, 1.06) | 0.92 (0.80, 1.07) |
| Soybean |  |  |  |  |
| < median | 2,544/166,650 | 1.00 (Ref.) | 1.00 (Ref.) | 1.00 (Ref.) |
| ≥ median | 138/11,498 | 0.78 (0.66, 0.93) | 0.89 (0.75, 1.06) | 0.89 (0.75, 1.05) |
| Whole grains |  |  |  |  |
| < median | 1,420/89,465 | 1.00 (Ref.) | 1.00 (Ref.) | 1.00 (Ref.) |
| ≥ median | 1,262/88,683 | 0.89 (0.83, 0.96) | 0.85 (0.79, 0.92) | 0.86 (0.80, 0.93) |
| High-fat diet |  |  |  |  |
| ≥ median | 185/13,653 | 1.00 (Ref.) | 1.00 (Ref.) | 1.00 (Ref.) |
| < median | 2,497/164,495 | 1.12 (0.97, 1.30) | 1.05 (0.90, 1.22) | 1.07 (0.92, 1.24) |
| Processed meat |  |  |  |  |
| ≥ median | 1,350/83,843 | 1.00 (Ref.) | 1.00 (Ref.) | 1.00 (Ref.) |
| < median | 1,332/94,305 | 0.88 (0.81, 0.95) | 0.92 (0.86, 1.00) | 0.92 (0.86, 1.00) |
| Red meat |  |  |  |  |
| ≥ median | 1,344/84,143 | 1.00 (Ref.) | 1.00 (Ref.) | 1.00 (Ref.) |
| < median | 1,338/94,005 | 0.89 (0.83, 0.96) | 0.97 (0.90, 1.04) | 0.96 (0.89, 1.04) |
| Refined grains |  |  |  |  |
| ≥ median | 1,326/88,276 | 1.00 (Ref.) | 1.00 (Ref.) | 1.00 (Ref.) |
| < median | 1,356/89,872 | 1.01 (0.93, 1.08) | 0.96 (0.88, 1.04) | 0.95 (0.89, 1.04) |

Abbreviations: CI, confidence interval; DI-GM, dietary index for gut microbiota; HR, hazard ratio; Ref, reference.

^1^ Model 1 was adjusted for age (continuous, year), sex (female or male), body mass index (continuous, kg/m^2^), and total energy intake (continuous, kJ/d).

^2^ Model 2 was further adjusted for annual household income (≥ 3100 pound or < 3100 pound), education level (high, medium, or no above), Townsend deprivation index (continuous), smoking status (never, previous, or current), alcohol consumption (never, previous, or current), family history of cancer (no or yes), and physical activity level (high, medium, or low), based on model 1

**Supplementary Table 9. Subgroup analysis for the association between DI-GM and risk of gastrointestinal cancer**

| **Subgroups** | **DI-GM [HR (95%CI) ^1^]** | | | | | | | ***P* for interaction** | |
| --- | --- | --- | --- | --- | --- | --- | --- | --- | --- |
|  | **Low** | | **Moderate** | | | **High** | |  |  |
| Age |  |  | | |  | | | < 0.001 | |
| < 60 years | 1.00 (Ref.) | | 0.94 (0.82, 1.08) | | | 0.97 (0.82, 1.15) | |  | |
| ≥ 60 years | 1.00 (Ref.) | | 0.92 (0.82, 1.02) | | | 0.81 (0.71, 0.93) | |  | |
| Sex |  |  | | |  | | | < 0.001 | |
| Female | 1.00 (Ref.) | | | 0.91 (0.79, 1.05) | | | 0.92 (0.78, 1.08) | |  |
| Male | 1.00 (Ref.) | | | 0.91 (0.81, 1.01) | | | 0.77 (0.67, 0.88) | |  |
| BMI ^2^ |  |  | | |  | | | | < 0.001 |
| Normal | 1.00 (Ref.) | | | 0.99 (0.83, 1.17) | | | 0.93 (0.77, 1.12) | |  |
| Overweight | 1.00 (Ref.) | | | 0.87 (0.77, 0.98) | | | 0.73 (0.62, 0.85) | |  |
| Obesity | 1.00 (Ref.) | | | 0.89 (0.75, 1.06) | | | 0.94 (0.75, 1.17) | |  |
| Education level |  |  | | |  | | | | 0.209 |
| No qualification | 1.00 (Ref.) | | | 0.93 (0.73, 1.18) | | | 0.84 (0.61, 1.17) | |  |
| Medium | 1.00 (Ref.) | | | 0.92 (0.81, 1.04) | | | 0.91 (0.78, 1.06) | |  |
| High | 1.00 (Ref.) | | | 0.89 (0.77, 1.02) | | | 0.76 (0.65, 0.90) | |  |
| Townsend deprivation index | |  | | |  | | | | 0.005 |
| < median | 1.00 (Ref.) | | | 0.80 (0.71, 0.90) | | | 0.74 (0.64, 0.86) | |  |
| ≥ median | 1.00 (Ref.) | | | 1.04 (0.92, 1.18) | | | 0.94 (0.81, 1.10) | |  |
| Smoking status |  |  | | |  | | | | < 0.001 |
| Never | 1.00 (Ref.) | | | 0.99 (0.87, 1.13) | | | 0.85 (0.73, 1.00) | |  |
| Previous | 1.00 (Ref.) | | | 0.83 (0.73, 0.94) | | | 0.79 (0.68, 0.92) | |  |
| Current | 1.00 (Ref.) | | | 0.94 (0.71, 1.25) | | | 1.05 (0.72, 1.52) | |  |
| Alcohol consumption |  |  | | |  | | | | 0.184 |
| Never | 1.00 (Ref.) | | | 0.73 (0.44, 1.21) | | | 0.56 (0.29, 1.07) | |  |
| Previous | 1.00 (Ref.) | | | 1.21 (0.76, 1.94) | | | 0.91 (0.52, 1.59) | |  |
| Current | 1.00 (Ref.) | | | 0.90 (0.82, 0.98) | | | 0.84 (0.75, 0.93) | |  |
| Family history of cancer |  |  | | |  | | | | 0.098 |
| No | 1.00 (Ref.) | | | 0.88 (0.79, 0.99) | | | 0.86 (0.75, 0.98) | |  |
| Yes | 1.00 (Ref.) | | | 0.95 (0.83, 1.09) | | | 0.81 (0.68, 0.95) | |  |
| Physical activity |  |  | | |  | | | | 0.206 |
| Low | 1.00 (Ref.) | | | 0.78 (0.63, 0.96) | | | 0.79 (0.60, 1.03) | |  |
| Medium | 1.00 (Ref.) | | | 0.93 (0.83, 1.04) | | | 0.82 (0.72, 0.94) | |  |
| High | 1.00 (Ref.) | | | 0.94 (0.78, 1.13) | | | 0.89 (0.72, 1.10) | |  |

Low: DI-GM ≤ 4; moderate: 5 ≤ DI-GM ≤ 6; high: DI-GM ≥ 7.

Abbreviations: BMI, body mass index; CI, confidence interval; DI-GM, dietary index for gut microbiota; HR, hazard ratio; Ref, reference.

^1^ Hazard ratio with 95% confidence interval was calculated using Cox regression models that were adjusted for age (continuous, year), sex (female or male), body mass index (continuous, kg/m^2^), total energy intake (continuous, kJ/d), annual household income (< 3100 pound or ≥ 3100 pound), education level (no above, medium, or high), Townsend deprivation index (continuous), smoking status (never, previous, or current), alcohol consumption (never, previous, or current), family history of cancer (no or yes), and physical activity level (low, medium, or high), unless a certain variable was the basis of the stratification.

^2^ BMI was divided into three levels: normal (< 24.9 kg/m^2^), overweight (25-30 kg/m^2^), and obesity (> 30 kg/m^2^).

**Supplementary Table 10. Sensitivity analyses for excluding participants with less than two years of follow-up**

| **DI-GM** | **Cases/Total** | **HR (95%CI)** | | |
| --- | --- | --- | --- | --- |
|  |  | **Crude model** | **Model 1 ^1^** | **Model 2 ^2^** |
| Low | 929/66,969 | 1.00 (Ref.) | 1.00 (Ref.) | 1.00 (Ref.) |
| Moderate | 939/68,620 | 0.99 (0.90, 1.08) | 0.94 (0.86, 1.03) | 0.95 (0.87, 1.05) |
| High | 522/42,267 | 0.89 (0.80, 0.99) | 0.85 (0.76, 0.95) | 0.86 (0.77, 0.97) |
| *P* for trend |  | 0.040 | 0.004 | 0.011 |
| Per SD increment |  | 0.97 (0.95, 0.99) | 0.96 (0.94, 0.98) | 0.96 (0.94, 0.98) |

Low: DI-GM ≤ 4; moderate: 5 ≤ DI-GM ≤ 6; high: DI-GM ≥ 7.

Abbreviations: CI, confidence interval; DI-GM, dietary index for gut microbiota; HR, hazard ratio; Ref, reference; SD, standard deviation.

^1^ Model 1 was adjusted for age (continuous, year), sex (female or male), body mass index (continuous, kg/m^2^), and total energy intake (continuous, kJ/d).

^2^ Model 2 was further adjusted for annual household income (< 3100 pound or ≥ 3100 pound), education level (no above, medium, or high), Townsend deprivation index (continuous), smoking status (never, previous, or current), alcohol consumption (never, previous, or current), family history of cancer (no or yes), and physical activity level (low, medium, or high), based on model 1.

**Supplementary Table 11. Sensitivity analyses for recalculating the DI-GM after including pulses intake to reflect chickpea**

| **DI-GM** | **Cases/Total** | **HR (95%CI)** | | |
| --- | --- | --- | --- | --- |
|  |  | **Crude model** | **Model 1 ^1^** | **Model 2 ^2^** |
| Low | 1,008/62,842 | 1.00 (Ref.) | 1.00 (Ref.) | 1.00 (Ref.) |
| Moderate | 1,012/67,571 | 0.99 (0.90, 1.08) | 0.94 (0.86, 1.03) | 0.95 (0.87, 1.05) |
| High | 662/47,735 | 0.89 (0.80, 0.99) | 0.85 (0.76, 0.95) | 0.86 (0.77, 0.97) |
| *P* for trend |  | 0.040 | 0.004 | 0.011 |
| Per SD increment |  | 0.93 (0.89, 0.97) | 0.91 (0.88, 0.95) | 0.92 (0.88, 0.96) |

Low: DI-GM ≤ 4; moderate: 5 ≤ DI-GM ≤ 6; high: DI-GM ≥ 7.

Abbreviations: CI, confidence interval; DI-GM, dietary index for gut microbiota; HR, hazard ratio; Ref, reference; SD, standard deviation.

^1^ Model 1 was adjusted for age (continuous, year), sex (female or male), body mass index (continuous, kg/m^2^), and total energy intake (continuous, kJ/d).

^2^ Model 2 was further adjusted for annual household income (< 3100 pound or ≥ 3100 pound), education level (no above, medium, or high), Townsend deprivation index (continuous), smoking status (never, previous, or current), alcohol consumption (never, previous, or current), family history of cancer (no or yes), and physical activity level (low, medium, or high), based on model 1.

**Supplementary Table 12. Sensitivity analyses for recalculating the DI-GM after excluding cranberries**

| **DI-GM** | **Cases/Total** | **HR (95%CI)** | | |
| --- | --- | --- | --- | --- |
|  |  | **Crude model** | **Model 1 ^1^** | **Model 2 ^2^** |
| Low | 626/37,073 | 1.00 (Ref.) | 1.00 (Ref.) | 1.00 (Ref.) |
| Moderate | 1126/74,443 | 0.89 (0.81, 0.99) | 0.85 (0.77, 0.94) | 0.86 (0.78, 0.95) |
| High | 930/66,632 | 0.82 (0.74, 0.91) | 0.77 (0.70, 0.85) | 0.78 (0.70, 0.87) |
| *P* for trend |  | <0.001 | <0.001 | <0.001 |
| Per SD increment |  | 0.96 (0.94, 0.98) | 0.95 (0.93, 0.97) | 0.95 (0.93, 0.97) |

Low: DI-GM ≤ 4; moderate: 5 ≤ DI-GM ≤ 6; high: DI-GM ≥ 7.

Abbreviations: CI, confidence interval; DI-GM, dietary index for gut microbiota; HR, hazard ratio; Ref, reference; SD, standard deviation.

^1^ Model 1 was adjusted for age (continuous, year), sex (female or male), body mass index (continuous, kg/m^2^), and total energy intake (continuous, kJ/d).

^2^ Model 2 was further adjusted for annual household income (< 3100 pound or ≥ 3100 pound), education level (no above, medium, or high), Townsend deprivation index (continuous), smoking status (never, previous, or current), alcohol consumption (never, previous, or current), family history of cancer (no or yes), and physical activity level (low, medium, or high), based on model 1.

**Supplementary Table 13. Sensitivity analysis for excluding participants who completed the questionnaire only once**

| **DI-GM** | **Cases/Total** | **HR (95%CI)** | | |
| --- | --- | --- | --- | --- |
|  |  | **Crude model** | **Model 1 ^1^** | **Model 2 ^2^** |
| Low | 647/40544 | 1.00 (Ref.) | 1.00 (Ref.) | 1.00 (Ref.) |
| Moderate | 621/40352 | 0.96 (0.86, 1.07) | 0.93 (0.83, 1.04) | 0.94 (0.84, 1.05) |
| High | 373/28133 | 0.83 (0.73, 0.94) | 0.81 (0.71, 0.92) | 0.82 (0.72, 0.93) |
| *P* for trend |  | 0.004 | 0.001 | 0.003 |
| Per SD increment |  | 0.96 (0.94, 0.98) | 0.95 (0.93, 0.98) | 0.96 (0.93, 0.98) |

Low: DI-GM ≤ 4; moderate: 5 ≤ DI-GM ≤ 6; high: DI-GM ≥ 7.

Abbreviations: CI, confidence interval; DI-GM, dietary index for gut microbiota; HR, hazard ratio; Ref, reference; SD, standard deviation.

^1^ Model 1 was adjusted for age (continuous, year), sex (female or male), body mass index (continuous, kg/m^2^), and total energy intake (continuous, kJ/d).

^2^ Model 2 was further adjusted for annual household income (< 3100 pound or ≥ 3100 pound), education level (no above, medium, or high), Townsend deprivation index (continuous), smoking status (never, previous, or current), alcohol consumption (never, previous, or current), family history of cancer (no or yes), and physical activity level (low, medium, or high), based on model 1.

**Supplementary Table 14. Sensitivity analyses for excluding participants with unknown or missing covariates data**

| **DI-GM** | **Cases/Total** | **HR (95%CI)** | | |
| --- | --- | --- | --- | --- |
|  |  | **Crude model** | **Model 1 ^1^** | **Model 2 ^2^** |
| Low | 842/52057 | 1.00 (Ref.) | 1.00 (Ref.) | 1.00 (Ref.) |
| Moderate | 759/51182 | 0.92 (0.83, 1.01) | 0.88 (0.80, 0.97) | 0.89 (0.80, 0.98) |
| High | 445/31717 | 0.86 (0.77, 0.97) | 0.83 (0.74, 0.94) | 0.85 (0.75, 0.96) |
| *P* for trend |  | 0.009 | 0.001 | 0.004 |
| Per SD increment |  | 0.97 (0.94, 0.99) | 0.96 (0.93, 0.98) | 0.96 (0.94, 0.98) |

Low: DI-GM ≤ 4; moderate: 5 ≤ DI-GM ≤ 6; high: DI-GM ≥ 7.

Abbreviations: CI, confidence interval; DI-GM, dietary index for gut microbiota; HR, hazard ratio; Ref, reference; SD, standard deviation.

^1^ Model 1 was adjusted for age (continuous, year), sex (female or male), body mass index (continuous, kg/m^2^), and total energy intake (continuous, kJ/d).

^2^ Model 2 was further adjusted for annual household income (< 3100 pound or ≥ 3100 pound), education level (no above, medium, or high), Townsend deprivation index (continuous), smoking status (never, previous, or current), alcohol consumption (never, previous, or current), family history of cancer (no or yes), and physical activity level (low, medium, or high), based on model 1.

**Supplementary Table 15. Association between genetic Risk and risk of gastrointestinal cancer**

| **Genetic Risk** | **Cases/Total** | **HR (95%CI)** | | |
| --- | --- | --- | --- | --- |
|  |  | **Crude model** | **Model 1 ^1^** | **Model 2 ^2^** |
| Low | 304/35,630 | 1.00 (Ref.) | 1.00 (Ref.) | 1.00 (Ref.) |
| Intermediate | 1,511/106,889 | 1.66 (1.47, 1.88) | 1.65 (1.46, 1.87) | 1.65 (1.46, 1.87) |
| High | 867/35,629 | 2.88 (2.53, 3.28) | 2.90 (2.55, 3.31) | 2.89 (2.53, 3.29) |
| *P* for trend |  | <0.001 | <0.001 | <0.001 |
| Per SD increment |  | 1.47 (1.42, 1.53) | 1.48 (1.43, 1.54) | 1,48 (1.42, 1.54) |

Low genetic risk corresponds to CPRS quintile 1, intermediate genetic risk to quintile 2-quintile 4, and high genetic risk to quintile 5.

Abbreviations: CI, confidence interval; DI-GM, dietary index for gut microbiota; HR, hazard ratio; Ref, reference; SD, standard deviation.

^1^ Model 1 was adjusted for age (continuous, year), sex (female or male), body mass index (continuous, kg/m^2^), and total energy intake (continuous, kJ/d).

^2^ Model 2 was further adjusted for annual household income (≥ 3100 pound or ≤ 3100 pound), education level (high, medium, or no above), Townsend deprivation index (continuous), smoking status (never, previous, or current), alcohol consumption (never, previous, or current), family history of cancer (no or yes), and physical activity level (high, medium, or low), based on model 1.

**Supplementary Table 16. The combined effects of DI-GM and genetic risk with risk of gastrointestinal cancer**

| **DI-GM** | **Cases/Total** | **HR (95% CI)** | | |
| --- | --- | --- | --- | --- |
|  |  | **Crude model** | **Model 1 ^1^** | **Model 2 ^2^** |
| High genetic risk |  |  |  |  |
| Low | 329/13,647 | 1.00 (Ref.) | 1.00 (Ref.) | 1.00 (Ref.) |
| Moderate | 341/13,802 | 1.02 (0.88, 1.19) | 0.97 (0.83, 1.13) | 0.98 (0.84, 1.14) |
| High | 197/8,180 | 1.00 (0.84, 1.19) | 0.95 (0.79, 1.13) | 0.96 (0.80, 1.15) |
| Intermediate genetic risk |  |  |  |  |
| Low | 612/40,192 | 0.63 (0.55, 0.72) | 0.62 (0.54, 0.70) | 0.62 (0.54, 0.71) |
| Moderate | 573/41,116 | 0.57 (0.50, 0.66) | 0.54 (0.47, 0.62) | 0.55 (0.48, 0.63) |
| High | 326/25,581 | 0.52 (0.45, 0.61) | 0.49 (0.42, 0.57) | 0.50 (0.42, 0.58) |
| Low genetic risk |  |  |  |  |
| Low | 128/13,270 | 0.40 (0.32, 0.49) | 0.39 (0.32, 0.48) | 0.39 (0.32, 0.48) |
| Moderate | 116/13,793 | 0.35 (0.28, 0.43) | 0.32 (0.26, 0.40) | 0.33 (0.27, 0.41) |
| High | 60/8,567 | 0.29 (0.22, 0.38) | 0.27 (0.21, 0.36) | 0.28 (0.21, 0.36) |
| *P* for trend |  | 0.005 | <0.001 | <0.001 |

Low genetic risk corresponds to CPRS quintile 1, intermediate genetic risk to quintile 2-quintile 4, and high genetic risk to quintile 5.

Low: DI-GM ≤ 4; moderate: 5 ≤ DI-GM ≤ 6; high: DI-GM ≥ 7.

Abbreviations: CI, confidence interval; DI-GM, dietary index for gut microbiota; HR, hazard ratio; Ref, reference.

^1^ Model 1 was adjusted for age (continuous, year), sex (female or male), body mass index (continuous, kg/m^2^), and total energy intake (continuous, kJ/d).

^2^ Model 2 was further adjusted for annual household income (≥ 3100 pound or < 3100 pound), education level (high, medium, or no above), Townsend deprivation index (continuous), smoking status (never, previous, or current), alcohol consumption (never, previous, or current), family history of cancer (no or yes), and physical activity level (high, medium, or low), based on model 1.

**Supplementary Table 17. The combined effects of DI-GM and genetic risk with risk of esophageal cancer**

| **DI-GM** | **Cases/Total** | **HR (95% CI)** | | |
| --- | --- | --- | --- | --- |
|  |  | **Crude model** | **Model 1 ^1^** | **Model 2 ^2^** |
| High genetic risk |  |  |  |  |
| Low | 40/13,302 | 1.00 (Ref.) | 1.00 (Ref.) | 1.00 (Ref.) |
| Moderate | 25/13,770 | 0.60 (0.37, 0.99) | 0.61 (0.37, 1.01) | 0.65 (0.39, 1.07) |
| High | 15/8,557 | 0.58 (0.32, 1.05) | 0.60 (0.33, 1.10) | 0.65 (0.36, 1.18) |
| Intermediate genetic risk |  |  |  |  |
| Low | 86/40,526 | 0.71 (0.48, 1.03) | 0.72 (0.50, 1.05) | 0.73 (0.50, 1.06) |
| Moderate | 74/41,151 | 0.60 (0.41, 0.88) | 0.59 (0.40, 0.87) | 0.62 (0.42, 0.92) |
| High | 29/25,212 | 0.38 (0.24, 0.61) | 0.40 (0.25, 0.65) | 0.43 (0.27, 0.70) |
| Low genetic risk |  |  |  |  |
| Low | 15/13,281 | 0.38 (0.21, 0.68) | 0.38 (0.21, 0.69) | 0.39 (0.21, 0.70) |
| Moderate | 28/13,790 | 0.67 (0.42, 1.09) | 0.67 (0.41, 1.08) | 0.70 (0.43, 1.14) |
| High | 6/8,559 | 0.23 (0.10, 0.55) | 0.25 (0.11, 0.58) | 0.27 (0.11, 0.63) |
| *P* for trend |  | <0.001 | 0.001 | 0.007 |

Low genetic risk corresponds to PRS quintile 1, intermediate genetic risk to quintile 2-quintile 4, and high genetic risk to quintile 5.

Low: DI-GM ≤ 4; moderate: 5 ≤ DI-GM ≤ 6; high: DI-GM ≥ 7.

Abbreviations: CI, confidence interval; DI-GM, dietary index for gut microbiota; HR, hazard ratio; Ref, reference.

^1^ Model 1 was adjusted for age (continuous, year), sex (female or male), body mass index (continuous, kg/m^2^), and total energy intake (continuous, kJ/d).

^2^ Model 2 was further adjusted for annual household income (≥ 3100 pound or < 3100 pound), education level (high, medium, or no above), Townsend deprivation index (continuous), smoking status (never, previous, or current), alcohol consumption (never, previous, or current), family history of cancer (no or yes), and physical activity level (high, medium, or low), based on model 1.

**Supplementary Table 18. The combined effects of DI-GM and genetic risk with risk of colorectal cancer**

| **DI-GM** | **Cases/Total** | **HR (95% CI)** | | |
| --- | --- | --- | --- | --- |
|  |  | **Crude model** | **Model 1 ^1^** | **Model 2 ^2^** |
| High genetic risk |  |  |  |  |
| Low | 290/13,577 | 1.00 (Ref.) | 1.00 (Ref.) | 1.00 (Ref.) |
| Moderate | 290/13,830 | 0.98 (0.83, 1.15) | 0.92 (0.78, 1.08) | 0.92 (0.78, 1.09) |
| High | 182/8,222 | 1.03 (0.86, 1.24) | 0.97 (0.80, 1.17) | 0.97 (0.81, 1.17) |
| Intermediate genetic risk |  |  |  |  |
| Low | 477/40,260 | 0.55 (0.48, 0.64) | 0.54 (0.47, 0.63) | 0.55 (0.47, 0.63) |
| Moderate | 465/41,132 | 0.53 (0.45, 0.61) | 0.49 (0.43, 0.57) | 0.49 (0.43, 0.57) |
| High | 252/25,497 | 0.46 (0.39, 0.54) | 0.42 (0.35, 0.50) | 0.42 (0.36, 0.50) |
| Low genetic risk |  |  |  |  |
| Low | 70/13,272 | 0.24 (0.19, 0.32) | 0.24 (0.19, 0.31) | 0.24 (0.19, 0.31) |
| Moderate | 83/13,749 | 0.28 (0.22, 0.36) | 0.26 (0.20, 0.33) | 0.26 (0.21, 0.33) |
| High | 43/8,609 | 0.23 (0.17, 0.32) | 0.21 (0.16, 0.29) | 0.21 (0.16, 0.30) |
| *P* for trend |  | 0.079 | 0.003 | 0.003 |

Low genetic risk corresponds to PRS quintile 1, intermediate genetic risk to quintile 2-quintile 4, and high genetic risk to quintile 5.

Low: DI-GM ≤ 4; moderate: 5 ≤ DI-GM ≤ 6; high: DI-GM ≥ 7.

Abbreviations: CI, confidence interval; DI-GM, dietary index for gut microbiota; HR, hazard ratio; Ref, reference.

^1^ Model 1 was adjusted for age (continuous, year), sex (female or male), body mass index (continuous, kg/m^2^), and total energy intake (continuous, kJ/d).

^2^ Model 2 was further adjusted for annual household income (≥ 3100 pound or < 3100 pound), education level (high, medium, or no above), Townsend deprivation index (continuous), smoking status (never, previous, or current), alcohol consumption (never, previous, or current), family history of cancer (no or yes), and physical activity level (high, medium, or low), based on model 1.

**Supplementary Table 19. Additive and multiplicative interaction of DI-GM and genetic risk on gastrointestinal cancer**

| **DI-GM** | **Genetic Risk** | | | | ***P* for multiplicative interaction** |
| --- | --- | --- | --- | --- | --- |
|  | **Low genetic risk** | | **Intermediate genetic risk** | |  |
|  | **RERI (95%CI)** | **AP (95%CI)** | **RERI (95%CI)** | **AP (95%CI)** |  |
| Low | 1.00 (Ref.) | 1.00 (Ref.) | 1.00 (Ref.) | 1.00 (Ref.) | 0.37 |
| Moderate | -0.04 (-0.11, 0.03) | -0.13 (-0.35, 0.08) | -0.05 (-0.12, 0.03) | -0.09 (-0.23, 0.05) |  |
| High | -0.08 (-0.16, 0.00) | -0.29 (-0.57, 0.00) | -0.09 (-0.16, -0.01) | -0.17 (-0.33, -0.01) |  |

Low genetic risk corresponds to CPRS quintile 1, intermediate genetic risk to quintile 2-quintile 4, and high genetic risk to quintile 5.

Low: DI-GM ≤ 4; moderate: 5 ≤ DI-GM ≤ 6; high: DI-GM ≥ 7.

Abbreviations: AP, attributable proportion; CI, confidence interval; DI-GM, dietary index for gut microbiota; HR, hazard ratio; Ref, reference; RERI, relative excess risk due to interaction.

The models adjusted for age (continuous, year), sex (female or male), body mass index (continuous, kg/m^2^), total energy intake (continuous, kJ/d), annual household income (≥ 3100 pound or ≤ 3100 pound), education level (high, medium, or no above), Townsend deprivation index (continuous), smoking status (never, previous, or current), alcohol consumption (never, previous, or current), family history of cancer (no or yes), and physical activity level (high, medium, or low).

**Reference**

1. Bycroft C, Freeman C, Petkova D, Band G, Elliott LT, Sharp K, et al. The UK Biobank resource with deep phenotyping and genomic data. Nature. 2018;562(7726):203-9. doi: 10.1038/s41586-018-0579-z.

2. Fernandez-Rozadilla C, Timofeeva M, Chen Z, Law P, Thomas M, Schmit S, et al. Deciphering colorectal cancer genetics through multi-omic analysis of 100,204 cases and 154,587 controls of European and east Asian ancestries. Nat Genet. 2023;55(1):89-99. doi: 10.1038/s41588-022-01222-9.

3. Sharma S, Tapper WJ, Collins A, Hamady ZZR. Predicting Pancreatic Cancer in the UK Biobank Cohort Using Polygenic Risk Scores and Diabetes Mellitus. Gastroenterology. 2022;162(6):1665-74 e2. doi: 10.1053/j.gastro.2022.01.016.

4. Bianco C, Jamialahmadi O, Pelusi S, Baselli G, Dongiovanni P, Zanoni I, et al. Non-invasive stratification of hepatocellular carcinoma risk in non-alcoholic fatty liver using polygenic risk scores. J Hepatol. 2021;74(4):775-82. doi: 10.1016/j.jhep.2020.11.024.

5. Choi J, Jia G, Wen W, Long J, Zheng W. Evaluating polygenic risk scores in assessing risk of nine solid and hematologic cancers in European descendants. Int J Cancer. 2020;147(12):3416-23. doi: 10.1002/ijc.33176.

6. Gharahkhani P, Fitzgerald RC, Vaughan TL, Palles C, Gockel I, Tomlinson I, et al. Genome-wide association studies in oesophageal adenocarcinoma and Barrett's oesophagus: a large-scale meta-analysis. Lancet Oncol. 2016;17(10):1363-73. doi: 10.1016/S1470-2045(16)30240-6.

7. Zhu M, Wang T, Huang Y, Zhao X, Ding Y, Zhu M, et al. Genetic Risk for Overall Cancer and the Benefit of Adherence to a Healthy Lifestyle. Cancer Res. 2021;81(17):4618-27. doi: 10.1158/0008-5472.CAN-21-0836.

8. Kase BE, Liese AD, Zhang J, Murphy EA, Zhao L, Steck SE. The Development and Evaluation of a Literature-Based Dietary Index for Gut Microbiota. Nutrients. 2024;16(7). doi: 10.3390/nu16071045.

9. Perez-Cornago A, Pollard Z, Young H, van Uden M, Andrews C, Piernas C, Key TJ, Mulligan A, Lentjes M. Description of the updated nutrition calculation of the Oxford WebQ questionnaire and comparison with the previous version among 207,144 participants in UK Biobank. Eur J Nutr. 2021;60(7):4019-30. doi: 10.1007/s00394-021-02558-4.

10. Piernas C, Perez-Cornago A, Gao M, Young H, Pollard Z, Mulligan A, et al. Describing a new food group classification system for UK biobank: analysis of food groups and sources of macro- and micronutrients in 208,200 participants. Eur J Nutr. 2021;60(5):2879-90. doi: 10.1007/s00394-021-02535-x.

11. Hua B, Dong Z, Yang Y, Liu W, Chen S, Chen Y, Sun X, Ye D, Li J, Mao Y. Dietary Carbohydrates, Genetic Susceptibility, and Gout Risk: A Prospective Cohort Study in the UK. Nutrients. 2024;16(17). doi: 10.3390/nu16172883.

12. Orliacq J, Pérez-Cornago A, Parry SA, Kelly RK, Koutoukidis DA, Carter JL. Associations between types and sources of dietary carbohydrates and liver fat: a UK Biobank study. BMC Med. 2023;21(1):444. doi: 10.1186/s12916-023-03135-8.
